# Block Length-Dependent Protein Fouling on Poly(2-oxazoline)-Based Polymersomes: Influence on Macrophage Association and Circulation Behavior

**DOI:** 10.1002/smll.202201993

**Published:** 2022-06-07

**Authors:** Adrian Najer, Alexis Belessiotis-Richards, Hyemin Kim, Catherine Saunders, Federico Fenaroli, Christopher Adrianus, Junyi Che, Renée L. Tonkin, Håkon Høgset, Samuel Lörcher, Matthew Penna, Stuart G. Higgins, Wolfgang Meier, Irene Yarovsky, Molly M. Stevens

**Affiliations:** Department of Materials, Department of Bioengineering and Institute of Biomedical Engineering, Imperial College London, London SW7 2AZ, UK; Department of Biosciences, University of Oslo, Blindernveien 31, Oslo 0371, Norway; Department of Materials, Department of Bioengineering and Institute of Biomedical Engineering, Imperial College London, London SW7 2AZ, UK; Department of Chemistry, University of Basel, Mattenstrasse 24a, BPR 1096, Basel 4058, Switzerland; School of Engineering, RMIT University, Melbourne, Victoria 3001, Australia; Department of Materials, Department of Bioengineering and Institute of Biomedical Engineering, Imperial College London, London SW7 2AZ, UK; Department of Chemistry, University of Basel, Mattenstrasse 24a, BPR 1096, Basel 4058, Switzerland; School of Engineering, RMIT University, Melbourne, Victoria 3001, Australia; Department of Materials, Department of Bioengineering and Institute of Biomedical Engineering, Imperial College London, London SW7 2AZ, UK

**Keywords:** atomistic simulations, nanoparticles, protein corona, protein fouling, zebrafish embryos

## Abstract

Polymersomes are vesicular structures self-assembled from amphiphilic block copolymers and are considered an alternative to liposomes for applications in drug delivery, immunotherapy, biosensing, and as nanoreactors and artificial organelles. However, the limited availability of systematic stability, protein fouling (protein corona formation), and blood circulation studies hampers their clinical translation. Poly(2-oxazoline)s (POx) are valuable antifouling hydrophilic polymers that can replace the current gold-standard, poly(ethylene glycol) (PEG), yet investigations of POx functionality on nanoparticles are relatively sparse. Herein, a systematic study is reported of the structural, dynamic and antifouling properties of polymersomes made of poly(2-methyl-2-oxazoline)-*block*-poly(dimethylsiloxane)-*block*-poly(2-methyl-2-oxazoline) (PMOXA-*b*-PDMS-*b*-PMOXA). The study relates in vitro antifouling performance of the polymersomes to atomistic molecular dynamics simulations of polymersome membrane hydration behavior. These observations support the experimentally demonstrated benefit of maximizing the length of PMOXA (degree of polymerization *(DP)* > 6) while keeping PDMS at a minimal length that still provides sufficient membrane stability *(DP* > 19). In vitro macrophage association and in vivo blood circulation evaluation of polymersomes in zebrafish embryos corroborate these findings. They further suggest that single copolymer presentation on polymersomes is outperformed by blends of varied copolymer lengths. This study helps to rationalize design rules for stable and low-fouling polymersomes for future medical applications.

## 1 Introduction

Polymer-based vesicles, called polymersomes,^[1]^ are promising nanomaterials with potential biomedical applications in drug delivery, immunotherapy, biosensing, and as nanoreactors and artificial organelles in vitro and in vivo.^[2–7]^ However, clinical translation of polymersomes and, more broadly, many other nanomedicines is hampered due to nano-bio interactions in biological environments.^[3,8]^ These interactions with biological fluids (e.g., blood serum) change various nanoparticle properties such as stability, release behavior, cell uptake, and biodistribution.^[8]^ This complex process of non-specific coating of nanoparticles by serum proteins is also known as protein fouling or protein corona formation. Surface binding of proteins is highly dependent on nanoparticle physicochemical properties such size, shape, curvature, surface chemistry and roughness, charge, and hydrophilicity.Extensive protein fouling (e.g., opsonins) can often be linked to high macrophage association/uptake (opsonization), shortened blood circulation times and vice versa.^[10–12]^ Recognition of nanoparticles by the immune system can also cause nanoparticle-directed immune responses that reduce therapeutic efficacy.^[13]^ In addition, nanoparticles can induce inflammatory responses, for example, through the complement system activation route, which can potentially lead to unwanted side effects but can also be beneficial in certain cases when fighting infections, in immunotherapy and vaccines.^[8]^ These examples highlight the need to minimize nano-bio interactions in most cases, however, recent data suggests that the low-fouling behavior of nanoparticles needs to be balanced with the ability to attract favorable serum components, such as clusterin, to, for example, extend blood circulation times.^[14–16]^ Yet, this behavior may not be true of all nanoparticles, since some exhibit lengthy circulation periods despite the absence of a protein corona.^[17]^ Finding the balance between antifouling and adsorption of favorable components to prolong blood circulation times, combined with the need to then enable interactions once at the target site, are key challenges in the field.^[18]^ Among the few existing polymersome studies, many of the trends observed for other nanoparticle systems have been confirmed. Even small changes, for example, in polymer chemical nature or length (degree of polymerization, *DP)*, can dramatically change antifouling behavior, which can have either a detrimental or beneficial impact on polymersome properties.^[19–21]^ In addition, other characteristics such as polymer flexibility, dispersity (*Đ*), glass transition temperature, membrane fluidity, surface density, and the nature of the polymer end group could all potentially influence protein interactions on polymersome surfaces.

To counteract the adsorption of massive amounts of protein, nanoparticles are often covered with highly hydrated, hydrophilic polymers, the most prominent example being polyethylene glycol (PEG). Polymer length and density are key for achieving antifouling function, however, even small changes can have a big impact, necessitating careful evaluation of each system.^[11,22–24]^ Despite some evidence of PEG-directed immune responses, PEG remains a safe formulation stabilizer, for example, as part of the RNA-based COVID-19 vaccines, and can endow nanomedicines with long-circulation behavior.^[25,26]^ Nevertheless, anti-PEG antibodies can complicate the efficacy of PEG-containing therapeutics, especially during the repeated administration. In summary, some drawbacks of PEG-based systems include the possibility to stimulate anti-PEG IgM antibody responses, allergic reactions in some people, and the potential to produce toxic side products through oxidative degradation.^[27]^ Hence, finding alternative polymers with similar antifouling properties is required.^[28]^ Poly(2-oxazoline)s (POx), including poly(2-methyl-2-oxazoline) (PMOXA), are widely recognized as viable PEG alternatives.^[27,29,30]^ Experimentally, PMOXA-based coatings have shown high hydration and low biofouling comparable to, or even better than, PEG-coatings.^[29]^ Further advantages of POx are their structural modularity and synthetic versatility.^[27]^

Besides experimental evaluations, computational methods are essential to achieve a more rational approach for engineering nanoparticles for drug delivery.^[31]^ Atomistic molecular dynamic simulation studies have suggested some different mechanisms for PEG and POx antifouling behavior, while the degree of hydration has been shown to play a key role in performance of both ligands.^[32–36]^ The recent theoretical simulations identified a significant role of the entropic penalty for protein adsorption induced by the antifouling ligand flexibility,^[32–34]^ as well as the dynamics of water molecules within the ligand bound hydration layer.^[35]^ They demonstrated that compared to the PEG chains, PMOXA ligands are less prone to the hydrophobic collapse while being generally less dynamic.^[33]^ Further, the simulations identified a key role of the antifouling ligand flexibility, related to the degree of polymerization as well as their spatial packing (i.e., ligand immobilization density), in providing the protein adsorption resistance.^[32,34]^ The most essential finding of the theoretical modelling was that for the varying chemical makeup of the antifouling ligands there exists an optimal ligand length and packing density that provides the degree of hydration and molecular dynamics required to maximize both the enthalpic and entropic penalties for non-specific protein adsorption.^[32]^

Relatively few experimental studies exist of POx behavior in nanomedicines such as liposomes^[37]^ and other nanoparticles,^[38,39]^ compared to the PEG literature.^[18]^ Regarding biomedical applications, many studies have shown the potential of POx-based nanoparticles for biosensing, drug delivery in cancer therapy, and as nanoreactors and artificial organelles.^[4,6,27]^ Systematic studies of nanoparticle surface interactions and downstream effects on macrophage uptake and blood circulation time as a function of surface chemistries, polymer lengths, and architecture are required to allow POx to be translated into therapeutics.^[3]^ PEGylated liposomes are still the gold-standard in the nanomedicine field, hence comparison to these nanoparticles is important for translational reasons.^[3]^ Here, we have intentionally used a zebrafish embryo model and analysis technique that was employed and developed previously to study the circulation behavior of PEGylated liposomes,^[40]^ to allow us to make comparisons between the two systems. The zebrafish embryo model allows high-throughput blood circulation analysis, generating data shown to correlate well with rodent models.^[40–44]^ Although there is some anecdotal evidence that poly(2-methyl-2-oxazoline)-*block*-poly(dimethylsiloxane)-*block*-poly(2-methyl-2-oxazoline) (PMOXA--*b*-PDMS-*b*-PMOXA)-based polymersomes are rapidly cleared from the bloodstream (within ≈2 h),^[45,46]^ there is a lack of comprehensive studies of polymer-some protein fouling, macrophage association, and blood circulation times.

Here, we provide a systematic polymersome stability and protein fouling study using a copolymer library of PMOXA-*b*-PDMS-*b*-PMOXA and including blends of copolymers with varied hydrophilic block lengths. The polymersomes were characterized by various complementary techniques, including electron microscopy and single-particle automated Raman trapping analysis (SPARTA). We then used a fluorescence correlation spectroscopy (FCS)-based method to study serum protein interaction with polymersomes in situ. The experimental findings were further related to atomistic molecular dynamic simulations. Combining in vitro and in silico techniques allows us to link the atomistic origin of antifouling behavior in these polymersomes to their performance in complex environments. We studied macrophage association in vitro and blood circulation behavior in zebrafish embryos for the most promising formulations. The detailed understanding gained herein will allow the design of polymersomes with even greater biofouling resistance for future applications in vivo.

## 2 Results and Discussion

### 2.1 Polymersome Assembly and Characterization

**Figure 1:**
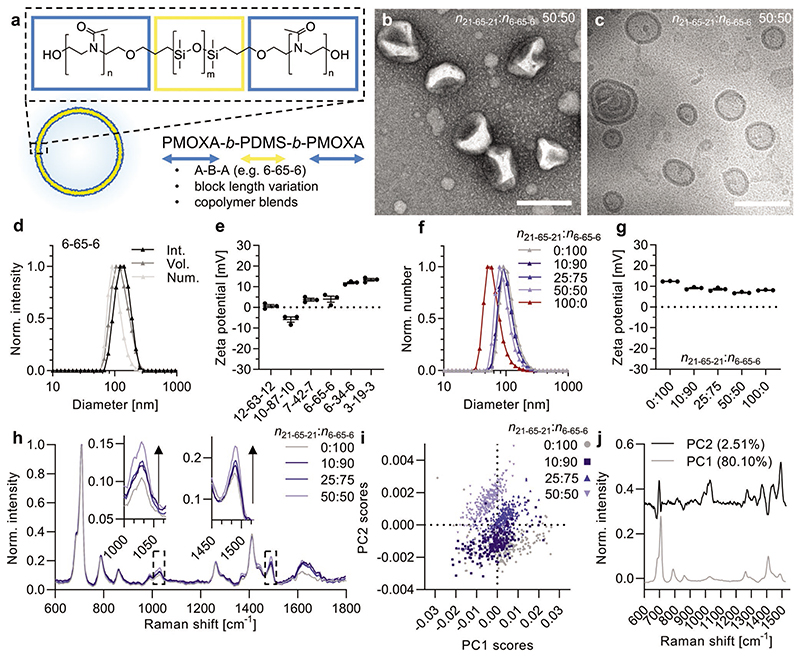
Polymersome characteristics with respect to block lengths and copolymer blend ratios. a) Schematic of chemical structure of PMOXA-*b*-PDMS-*b*-PMOXA and polymersome assembly (numbers, e.g., 6-65-6, correspond to *DP* of each block forming these ABA type copolymers). b) TEM image of polymersomes made from a 50 mol% blend of 21-65-21 and 6-65-6 (*n*_21-65-21_:*n*_6-65-6_ 50:50). Scale bar, 200 nm. c) Cryo-TEM image of (b). Scale bar, 200 nm. d) DLS measurement of 6-65-6 polymersomes (mean of technical triplicates; Int., Vol., and Num. are intensity, volume, and number distributions, respectively). e) Zeta potential measurements of various polymersomes (mean ± s.e.m., technical triplicates). f) DLS measurement (number distribution) of assemblies made from 6-65-6, 21-65-21, and blends (mean of technical triplicates). g) Zeta potential measurements of samples in (f) (mean ± s.e.m., technical triplicates). h) SPARTA data showing average Raman spectra for the blends in (f) (*n* > 200 traps each). All data was normalized to the main PDMS peak (Si–C at 708 cm^−1^) to compare the variations in PMOXA content. Zooms highlight PMOXA-related peaks increasing with higher mole fraction of the long copolymer 21-65-21 within the blends. i) PCA of data shown in (h) with each dot representing one nanoparticle trap. j) PC1 and PC2 scores of panel (i) showing that PC1 mainly corresponds to PDMS-related Raman peaks, while PC2 represents mainly PMOXA-related peaks. The percentage values refer to the variance between samples that is captured by each PC.

PMOXA-*b*-PDMS-*b*-PMOXA copolymers were employed as polymersome building blocks (Figure 1a) due to their widespread use in studies including drug delivery, as nanoreactors, and artificial organelles. We analyzed the influence of relative PMOXA and PDMS lengths and underlying membrane dynamics as well as copolymer blends of various lengths on stability and protein fouling using our copolymer library (see Table 1).^[41–43]^ Names of these ABA type copolymers represent *DP* of the corresponding blocks (e.g., PMOXA_6_-*b*-PDMS_65_-*b*-PMOXA_6_ is abbreviated as 6-65-6) and blends are denoted by their molar ratios (e.g., 50:50 *n*_21-65-21_:*n*_6-65-6_ is a mix of equal moles of each copolymer). Polymersomes were assembled using the thin film rehydration and extrusion (through 100 nm-pore membranes) technique. The obtained polymersomes were characterized to be ≈100 nm in diameter, as measured by electron microscopy and dynamic light scattering (DLS, Figure 1b–d). The extrusion step ensured that all the vesicle-forming copolymers yielded similarly sized polymersomes, removing size as a confounding variable from our study.

Following the theoretical suggestions that surface density of PMOXA is highly important with respect to their antifouling behavior,^[33]^ we studied blends of copolymers with a constant hydrophobic block length (65 DMS units) but varied hydrophilic block length (here 6 and 21 units of MOXA, respectively). This will introduce a spacing between the overhanging 15 MOXA units depending on the molar ratios between the two copolymers 21-65-21 and 6-65-6, respectively (tested from 10 to 50 mol%). Copolymers with hydrophilic fractions *f*_hydrophilic_ = 35 ± 10% (weight ratio of hydrophilic blocks to full copolymer) are known to have the potential to form polymer-somes rather than micellar structures.[50] The long copolymer 21-65-21 (*f*_hydrophilic_ = 43%) used in our blends was intentionally chosen to be at the higher end of theoretical vesicle-forming hydrophilic fractions. Indeed, this copolymer alone did not efficiently form polymersomes (Figure 1f and Figure S1, Supporting Information). In contrast, blends of this long copolymer with up to 50:50 (*n*_21-65-21_:*n*_6-65-6_) of a vesicle-forming copolymer with a minimal PMOXA length (6-65-6, *f*_hydrophilic_ = 20%) revealed effective vesicle formation.

The hydrophilic fluorescent dye sulforhodamine B (SRB) was encapsulated in the polymersome’s aqueous core at constant molar polymer concentrations. After size exclusion chromatography (SEC) this provided a simple measure of relative polymersome formation efficacy and demonstrated that 21-65-21 alone did not entrap significant amounts of SRB, hence, did not efficiently assemble into polymersomes (Figure S1, Supporting Information). We also included characterization by transmission electron microscopy (TEM) and cryo-TEM (Figure 1b,c and Figure S1, Supporting Information) for the assemblies that we have not yet characterized in detail in our previous studies (6-65-6, 21-65-21, and blends).^[41–43]^ This analysis confirmed that 6-65-6 and the 50:50 (*n*_21-65-21_:*n*_6-65-6_) blend successfully formed polymersomes, while 21-65-21 mainly formed micelles of various morphologies. Zeta potentials for all the tested polymersomes were around neutral values, while there was a clear trend toward the slightly positive side with decreasing numbers of MOXA units in the hydrophilic block (Figure 1e,g). PMOXA is expected to provide neutral charge. The inability of short chain PMOXA to completely shield the surface, possibly allowing exposure of PDMS islets, might be a possible reason. PMOXA chains are known to be less dynamic and they do not readily collapse onto the surface compared to PEG.^[33]^

To establish whether blending the two copolymers 21-65-21 and 6-65-6 indeed provided polymersome membranes with increased PMOXA content, we employed SPARTA, a recently developed label-free Raman spectroscopy technique.^[51]^ This analysis is based on automated trapping of more than 200 individual polymersomes per sample, while acquiring Raman spectra for each single particle. The average Raman spectra for all the combined traps (Figure 1h) reveal characteristic peaks for PDMS (Si–C at 708 cm^−1^ and C–H_3_ at 1411 cm^−1^) and PMOXA (1030 and 1490 cm^−1^). The broad signal ≈1635 cm^−1^ is mainly residual water Raman signal, and the variation here derives from small differences in background subtraction. The data was normalized to the main PDMS peak (Si–C at 708 cm^−1^) to compare differences in relative PMOXA content, independent of vesicle size. As expected, the PMOXA-related Raman peaks increased with increasing mole fraction of 21-65-21 within the blends. Plotting all the individual traps shows single populations that spread around a mean value (Figure S2, Supporting Information). Further, principal component analysis (PCA) successfully distinguished populations based on their PMOXA content (Figure 1i,j). The principle components (PC) from a 2 component principal component analysis (PCA) model resembled PDMS (PC1) and PMOXA (PC2) spectra, respectively (Figure 1j).^[52]^ Plotting the component scores for each particle from the 4 different blends resolved polymersomes prepared with different mole fractions of 21-65-21 (Figure 1i,j), confirming the formation of distinct vesicle populations that incorporated both copolymers. The SPARTA analysis confirmed on a single-particle basis that copolymer blending resulted in polymersomes containing a blend of copolymers, rather than two separate populations of particles. This was expected due to the identical chemical nature of the two block copolymers. However, the chance that the copolymers of different lengths phase-separated within the membrane cannot be excluded.

### 2.2 Polymersome Stability and Antifouling Behavior

**Figure 2:**
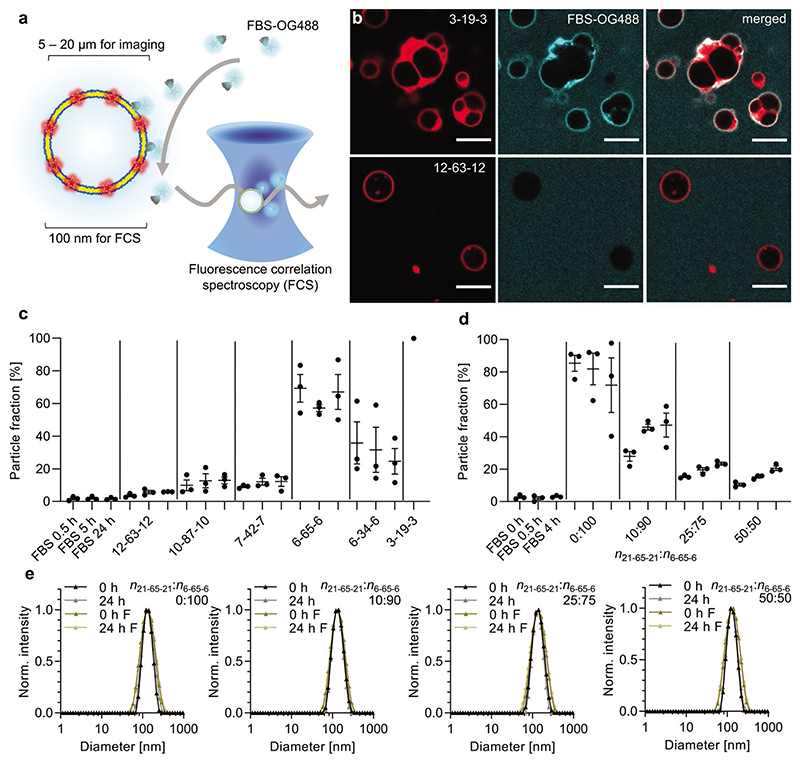
Protein fouling and stability study of polymersomes. a) Schematic of polymersome serum protein interaction study using fluorescence imaging (microscale) and FCS (nanoscale). b) Binding of randomly labeled fetal bovine serum (FBS-OG488) to giant microscaled polymersomes made from copolymers 3-19-3 and 12-63-12, respectively, after 4 h incubation at 37 °C. Red channel shows membrane stain and cyan is FBS-OG488. Scale bars, 10 μm. c,d) Median particle fractions from two-component fits of FCS curves (example curves and fitting shown in Figure S6, Supporting Information) after mixing c) unlabeled polymersomes or d) unlabeled polymersomes made from 21-65-21:6-65-6 blends with FBS-OG488, revealing extent of protein fouling on polymersome surface (mean ± s.e.m. of *N* = 3 independent experiments with *n* = 25 technical repeats each, see Figure S6 and S7, Supporting Information, for individual data sets). High particle fractions indicate high protein binding, while low particle fractions show low protein fouling. Only the first timepoint is shown for 3-19-3 in (c), since FBS-OG488 addition caused aggregation in this sample. e) DLS measurement (intensity distributions) of assemblies made from 6-65-6 and blends (mean of technical triplicates) after incubation in PBS or 10 v/v% FBS (abbreviated F in plot legends) at 37 °C, after mixing and 24 h later.

We next sought to determine the antifouling behavior of our polymersome library. In general, excessive protein binding can be considered detrimental in terms of blood circulation behavior in vivo.^[10–12]^ Due to the difficulties in studying protein binding to water-filled nanoparticles, such as liposomes and polymersomes, with standard techniques,^[53]^ we employed methods that allow in situ protein corona detection without purification. For initial visualization of the protein-polymer membrane interactions we employed confocal fluorescence microscopy (Figure 2a). First, we formed giant microscale polymersomes for the two copolymers with the longest and shortest hydrophilic PMOXA blocks (12-63-12 and 3-19-3, see Table 1). After sedimentation of the giant polymersomes in a glass chamber, randomly labeled fetal bovine serum (FBS-OG488, free amines labeled via amine-reactive NHS-OG488) was added and incubated at 37 °C for 4 h. Confocal fluorescence imaging revealed binding of FBS-OG488 (cyan) to 3-19-3 (red, hydrophobic membrane stain Bodipy630), while 12-63-12 showed no apparent FBS-OG488 rings around the giant polymersomes (Figure 2b, and Figures S3 and S4, Supporting Information).

Fluorescence imaging provided a valuable qualitative overview for these two most extreme cases of hydrophilic block lengths. However, it does not allow quantitative comparison between nuanced differences in protein corona formation, caused by intermediate PMOXA lengths and blends. This is exemplified by images of 6-65-6 giant polymersomes with FBS-OG488, which showed very little and non-quantifiable protein binding by in situ confocal imaging (Figure S5, Supporting Information) but increased protein fouling versus 12-63-12 in our more sensitive and quantitative analysis by FCS below. Most biomedical applications of polymersomes also require diameters at the nanoscale, which can potentially influence their behavior in complex biological environments, including changes in protein corona formation, due to the increased curvature compared to giant polymersomes. Hence, we employed FCS to study non-specific protein adsorption on nanoscale polymersomes (extruded to 100 nm, see Figure 1) and using the entire polymer library (Figure 2a,c,d, and Figures S6 and S7, Supporting Information). FCS is a highly sensitive method for detecting nanoparticle loading/release, surface functionalization, enzyme kinetics and protein binding, including measurement of in situ protein corona formation (no purification needed).^[54–57]^

Analogous to the fluorescence imaging experiments (Figure 2b, and Figures S3–S5, Supporting Information), we studied protein fouling on nanoscale polymersomes (≈100 nm in diameter) using FCS by measuring mixtures of non-labeled polymersomes with randomly labeled FBS-OG488.^[57]^ When measuring mixtures of fast and slow diffusing species of varying brightness, corresponding FCS curves are dominated by bright and slow diffusing species.^[58]^ Therefore, even low levels of protein binding will be detected in our case since we have put the label on the fast-diffusing species (protein). In contrast to studies with defined labeled proteins such as BSA/ HSA, which require prior knowledge about the identity of the nanoparticle binding proteins, our approach does not require establishing any previous protein corona information. Our FCS method does not provide compositional information for the protein corona, but it allows rapid in situ measurement of corona formation on challenging light nanoparticles (aqueous core) in a biologically most relevant mixture of proteins (serum). This delivers qualitative and quantitative data in terms of general antifouling behavior. We can draw important comparisons between different types of nanoparticles, or even between very similar assemblies that only vary marginally, for example, in the *DP* of underlying copolymers.

Using two-component fits of the autocorrelation curves (Figure S6, Supporting Information), one fixed to free protein, the other to nanoparticle diffusion times, allows direct determination of the nanoparticle fraction over time (particle fraction [%], *F*2, see Experimental Section, Supporting Information). Our FCS analysis revealed a dependence of PMOXA-length with respect to non-specific protein binding, which becomes apparent when ordering the copolymers according to their PMOXA length (Figure 2c and Figure S6, Supporting Information). Longer PMOXA chains exposed on the polymersomes resulted in better protein repelling, as evidenced by a lower particle fraction obtained from the two-component fits of the autocorrelation curves. The cut off for achieving low particle fractions, and hence low protein binding, was between 6 and 7 MOXA units. The shortest copolymer 3-19-3 formed polymersomes that were unstable and aggregated upon FBS-OG488 addition (which caused sedimentation over time), therefore, only the first timepoint is shown. Short PMOXA chains might cause exposure of hydrophobic PDMS patches when considering a curved membrane in polymersomes. These hydrophobic patches might attract significant amounts of protein. This could also explain the apparently lower protein binding on lower curvature giant polymersomes (6-65-6 copolymer, Figure S5, Supporting Information) when compared to the higher protein binding on nanoscale polymersomes made from the same copolymer (Figure 2c). On the other hand, decreasing the PDMS length, which means increasing the speed of membrane lateral diffusion and decreasing membrane thickness,^[47]^ had an inverse relationship, 6-34-6 had slightly lower particle fractions than 6-65-6. The average counts per particle (CPP in kilohertz for the first time point: 3.2 for FBS-OG488, 3.6 for 12-63-12, 4.7 for 10-87-10, 3.9 for 7-42-7, 11.1 for 6-65-6, 8.8 for 6-34-6, and 135 for 3-19-3) from the average FCS autocorrelation curve analysis revealed much higher CPP for 3-19-3, hence confirming high protein binding versus all other samples as seen in the images (Figure 2b). This also explains the difficulty of observing smaller differences, for example, for 12-63-12 versus 6-65-6, by imaging (Figures S4 and S5, Supporting Information).

For the blends and when incorporating increasing amounts of 21-65-21 within 6-65-6-based polymersomes, a gradual decrease of the particle fraction was observed, confirming the better antifouling behavior of the blends with high 21-65-21 content (Figure 2d and Figure S7, Supporting Information). This demonstrates how formulations of vesicle-forming copolymers with relatively short PMOXA chains can be improved by blending in non-vesicle-forming copolymers with long PMOXA chains. This is important since PMOXA cannot be increased independently of PDMS, due to the limitation of *f*_hydrophilic_ of ≈35 ± 10%^[50]^ for vesicle formation. When using single length copolymers for vesicle formation, a longer PMOXA block always requires a longer PDMS block. This increases the hydrophobic membrane thickness and slows down lateral mobility within the membrane,^[47]^ increasing protein fouling and thereby counteracting the benefit of the longer PMOXA chain (Figure 2c). Our solution of blending copolymers of different lengths represents one option to increase PMOXA surface exposure, while keeping the hydrophobic membrane thickness the same.

Alternative to measuring binding of randomly labeled FBS-OG to our polymersomes, FCS also allows studying interactions with single labeled proteins in isolation. Due to the known importance of clusterin (apolipoprotein J) binding to nanoparticles for reducing macrophage uptake and extending blood circulation time,^[14–16]^ we additionally tested clusterin-OG binding to our polymersomes by FCS (Figure S7, Supporting Information). First, we confirmed successful labeling of clusterin by revealing a hydrodynamic diameter of 1.2 ± 0.1 nm for OG488 and 7.1 ± 1.3 nm for clusterin-OG by FCS autocorrelation analysis (3/25 curves removed from average due to some aggregates measured, see high particle fraction in Figure S7, Supporting Information). Interestingly, after incubation of polymersomes with clusterin-OG in phosphate buffered saline (PBS) for 7 h, all samples from the blend series showed similar particle fractions, indicating similar amounts of clusterin binding. In contrast, the serum data (FBS-OG) revealed higher binding of random serum components to 6-65-6 compared to the blends (Figure 2d). Hence, in a competitive scenario (full serum), the 50:50 blend-based polymersomes might adsorb relatively more clusterin than 6-65-6 (0:100) polymersomes. This could explain the lower macrophage uptake and longer circulation time of the 50:50 versus the 0:100 sample as presented in the last section.

Polymersome stability was also studied by DLS after incubating particles at 37 °C in either PBS or 10 v/v% FBS (Figure 2e and Figure S2, Supporting Information). We observed no changes in polymersome diameter, nor any aggregation, for the majority of our formulations. The clear exception was the variant 3-19-3, which increased in size when incubated in FBS and was also unstable in PBS after long-term incubation (Figure S2, Supporting Information). Our findings suggest that the minimum PDMS length, hence, minimum membrane thickness to provide high stability polymersomes necessary for biomedical applications lies somewhere in between 19 and 34 DMS units (6.0–9.2 nm^[47]^ membrane thickness). In terms of PMOXA length, a minimum of 7 units seems necessary for achieving low protein fouling. In the future, it would also be interesting to utilize our blending strategy to evaluate whether 3-19-3 polymersomes could be stabilized by blending in a copolymer with the same number of DMS repeating units (19) but much longer PMOXA chains. These longer hydrophilic chains could potentially help to minimize PDMS exposure and hence reduce aggregation.

### 2.3 Atomistic Simulations of Polymersome Membranes and Interfacial Water

**Figure 3:**
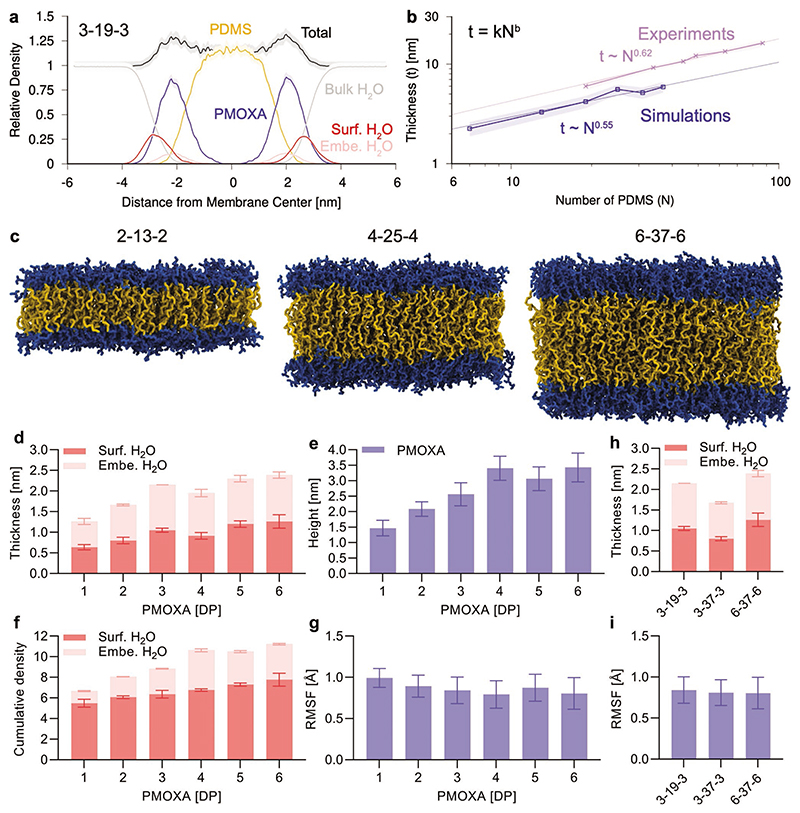
Atomistic molecular dynamics simulations of polymersome membranes. a) Cross-sectional densities of the membrane components showing PMOXA, PDMS, interfacial water, and bulk water (lines are means, shadings are s.d., data for the other simulated copolymers is shown in Figure S9, Supporting Information). b) Membrane thicknesses as a function of PDMS number from simulations and cryo-TEM data of our previous study.^[47]^ c) Cross-section simulation snapshots of membranes showing PDMS in yellow and PMOXA in blue, water is removed for clarity (snapshots for all the simulated copolymers are shown in Figure S8, Supporting Information). Effect of membrane composition on d) thickness of the interfacial water layer at the interface between PMOXA and water, e) cross-over height from bulk water to PMOXA, and f) area-under-curve of density for interfacial layer, and g) root mean-squared fluctuations (RMSF) of PMOXA groups during simulation. For these plots, the corresponding *DP*s for PDMS with increasing PMOXA are: 7, 13, 19, 25, 31, and 37, respectively. Effect of having same number of PMOXA or same number of PDMS groups on h) water layer thickness and i) PMOXA RMSF (3-19-3 and 6-37-6 correspond to 3 and 6 in other plots, respectively). All values are mean ± s.d.

To understand the mechanism of the protein antifouling properties of our polymersomes we employed a theoretical computational approach to studying the structure and dynamics of the polymer membranes and the associated interfacial surface water at all-atom resolution.^[29]^ Such an approach focusing on the interfacial ligand and water properties rather than simulating the actual protein binding to surfaces, has recently demonstrated the water structuring at the surface to be a good predictor of the ligand antifouling properties, at least on surfaces.^[29]^ We constructed atomistic models of various PMOXA-*b*-PDMS-*b*-PMOXA membranes comprised of copolymers with the following relative polymer *DP*s: 1-7-1, 2-13-2, 3-19-3, 4-25-4, 5-31-5, 3-37-3, and 6-37-6 (Table 1, Figure 3, and Figures S8 and S9, Supporting Information). Due to computational limitations, we simulated relatively small fragments of the membrane using model copolymer lengths corresponding to the shortest copolymers employed in the actual experiments described above, copolymer dispersity was not yet included (experimentally always > 1), and the simulations only provide limited information on conformation and dynamics of the ABA polymer chains within the membranes, which possibly include U- and I-shape configurations in reality.

Figure 3a shows the cross-sectional density of one of our PMOXA-*b*-PDMS-*b*-PMOXA membranes (3-19-3), highlighting the PDMS, PMOXA, and interfacial water types (data for remaining membranes in Figure S9, Supporting Information). We can clearly see the “dry” PDMS region where no water is present, within the membrane core, while “wet” PMOXA groups are in contact with interfacial water. Comparison of the thickness of our simulated membranes to experimental measurements performed in our previous work^[41]^ shows that simulations closely reproduced the experimental trends (Figure 3b). Selected snapshots of the simulated membranes with PDMS groups highlighted in yellow and PMOXA in blue are provided in Figure 3c and Figure S8, Supporting Information. Examining the membrane surface properties, we can see that the thickness of the interfacial water layer increases with the length of the PMOXA groups as well as the cross-over height at which PMOXA and bulk water interface, as expected (Figure 3d,e). This suggests that the longer the PMOXA chain, the further away the water layer spreads out from the PDMS core, creating a longer separation distance to cross by adsorbing proteins to foul the membrane. We can also see that the density of this interfacial water is higher for longer PMOXA chains again reinforcing the notion of a more challenging interface for fouling proteins to cross (Figure 3f). Comparatively, the RMSF of PMOXA chains is constant across the chain length, revealing similar dynamics of the chains, which suggests that the main changes between membranes are related to the structure and dynamics of the water layer at the interface. This lack of change is also noted for different PDMS lengths (Figure 3i). Finally, examining the 3-19-3, 3-37-3, and 6-37-6 membrane data in Figure 3h, we can see that the water layer thickness of the 3-37-3 case is lower than that of the 3-19-3 and the 6-37-6 cases, despite the same number of PMOXA and PDMS groups, respectively. This result points to a ratio of PMOXA to PDMS which can potentially optimize the antifouling ability of such membranes, in agreement with the experimental data shown above and the theoretical suggestions.^[32–34]^ Therefore, our all-atom polymersome simulations provided not only the structural insights but also the important baseline data for future comparative studies to optimize the antifouling membrane design, both theoretically and experimentally.

Specifically, we showed that maximizing PMOXA and minimizing PDMS substantially increased the interfacial water thickness and density leading to improved antifouling behavior of the membrane. This suggests that our approach of blending different copolymers with the same PDMS but different PMOXA block lengths provide a valuable option for improving PMOXA-based polymersome performance in biomedical applications. Within our blends, the overhanging PMOXA chains can be further spaced out by modulating the molar ratio used. Our theoretical all-atom simulations have shown that lower density PMOXA might be more efficient for the antifouling performance compared to PEG where density is less important.^[29]^ In the non-polymersome literature, antifouling behavior is commonly improved by using cyclic versus linear POx polymers, while extending the backbone monomer length using poly(2-methyl-2-oxazine) (PMeOzi) to form more flexible polymers revealed even greater hydration and accompanied antifouling behavior than PMOXA and PEG.^[27]^ Polymersome design, antifouling materials performance studies and simulations with further copolymer blends with even longer PMOXA chains, evaluating more complex architectures of POx, and polymersomes based on PMeOzi might be interesting future research avenues.

### 2.4 Macrophage Association and Circulation Analysis of Polymersomes

**Figure 4:**
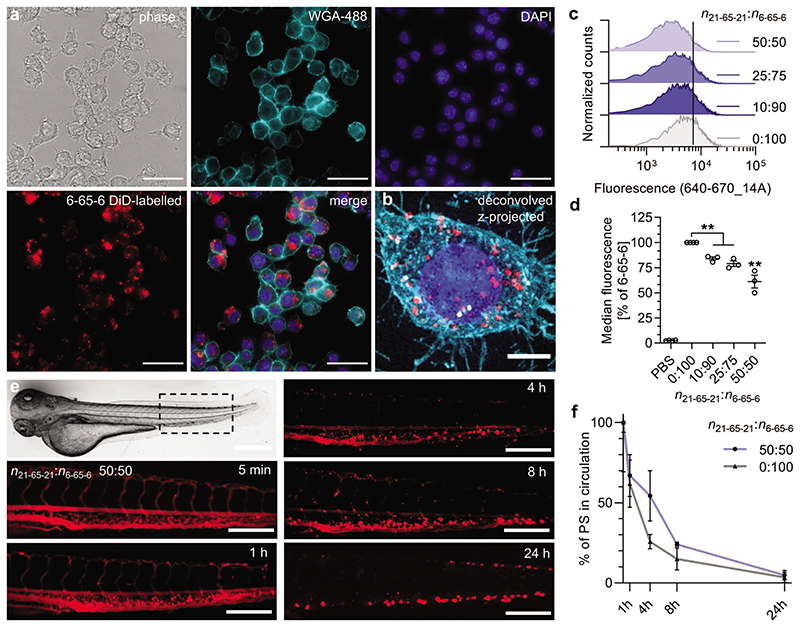
Cellular uptake and zebrafish embryo circulation study of polymersomes. a) Fluorescence widefield imaging of 6-65-6 polymersomes (red label, DiD) uptake in RAW 264.7 cells after 4 h incubation (cyan, WGA-488, membrane stain; blue, DAPI, nucleus). Scale bar, 30 μm. b) Zoom in of a z-projected deconvolved widefield fluorescence micrograph of a single cell, same sample as (a). Scale bar, 5 μm. c) Flow cytometry histograms for polymersome uptake in RAW 264.7 cells for the blend series 0:100–50:50 (*n*_21-65-21_:*n*_6-65-6_, DiD labeled) after 4 h incubation. The vertical line indicates the median fluorescence for 6-65-6 only (additional data for the series 12-63-12, 7-42-7 and 6-65-6 can be found in Figure S11, Supporting Information). d) Median fluorescence from data shown in panel (c) and independent repeats normalized to the 6-65-6 only sample. (*N* ≥ 3 independent experiments, one-way ANOVA with Tukey’s multiple comparisons test, *p < 0.05, **p < 0.01). e) Zebrafish embryo micrograph and representative fluorescence images of the tail area (indicated with dotted box) after administration of polymersomes made from the 50:50 blend (*n*_21-65-21_:*n*_6-65-6_, SRB loaded). Scale bars, 500 (brightfield) and 200 μm (fluorescence). Images for 0:100 can be found in Figure S12, Supporting Information. f) Quantitative analysis of percentage of polymersomes (PS) in circulation for 0:100 and 50:50 blends (*n*_21-65-21_:*n*_6-65-6_) studied over time (*n* = 5–8 embryos per sample and timepoint, mean ± s.d.).

Upon administration of nanoparticles into the bloodstream, a protein corona will form, eventually determining particle fate, for example, speed of elimination by macrophages. Opsonization, serum protein (opsonins) binding to nanoparticles and subsequent uptake by immune cells, such as macrophages, is a main elimination pathway for nanoparticles from the bloodstream.^[13]^ Macrophage association/uptake has previously been shown to be a good predictor of circulation time, with high and fast uptake correlating with short circulation times.^[10,11]^ We studied this correlation using the macrophage cell line RAW 264.7 and our polymersome library, choosing copolymers that formed stable polymersomes (see above) and only varied in the percentage of protein adsorption. Based on the FCS data (Figure 2c,d), we expected 6-65-6 polymersomes that bind high amounts of protein to undergo rapid uptake by macrophages (RAW 264.7 cell line), while 12-63-12 and 7-42-7 were expected to yield slower uptake. Widefield fluorescence microscopy of 6-65-6 polymersomes shows uptake in RAW cells (Figure 4a,b). Flow cytometry was next employed to quantify uptake in macrophages and to compare between different samples (Figure 4c,d and Figure S11, Supporting Information). This analysis revealed highest uptake for the 6-65-6 polymersomes as expected. When repeating the experiments with the copolymer blends, we found a clear trend of reduced uptake with increasing mole fraction of the long copolymer 21-65-21, which correlates with the FCS antifouling data (Figure 2d). The 50:50 (*n*_21-65-21_:*n*_6-65-6_) mixture provided the lowest uptake for all the samples studied. Example fluorescence images for uptake of 0:100 and 50:50 (*n*_21-65-21_:*n*_6-65-6_) polymersomes in RAW cells are shown in Figure S10, Supporting Information.

We further tested the worst (6-65-6) and the best polymersomes (50:50 blend, *n*_21-65-21_:*n*_6-65-6_) in terms of macrophage uptake in the zebrafish embryo model. The zebrafish analysis (Figure 4e,f and Figure S12, Supporting Information) showed good initial circulation for both polymersomes (1 h timepoint). For each subsequent timepoint they were gradually eliminated from the bloodstream. The observed dotted fluorescence in the caudal hematopoietic system (clearly visible from 4 h post injection) suggests that tissue-resident macrophages are actively removing the particles from the bloodstream. The 50:50 (*n*_21-65-21_:*n*_6-65-6_) blend-based polymersomes circulated slightly better (biggest difference at 4 h timepoint: 54 ± 16% versus 26 ± 4% in circulation, mean ± s.d.) than the 6-65-6 polymersomes (0 mol% blend), as predicted by our FCS protein binding, atomistic simulation, and macrophage uptake data. Analogously to studies in the PEG literature,^[14–16]^ the potentially higher relative clusterin binding for 50:50 versus 0:100 (*n*_21-65-21_:*n*_6-65-6_) (Figure 2d and Figure S7, Supporting Information) is a possible explanation for the lower macrophage uptake (Figure 4d) and extended circulation time for 50:50 (Figure 4f). Our polymersomes were almost completely eliminated within 24 h (4% and 5% left in circulation for 0:100 and 50:50 [*n*_21-65-21_:*n*_6-65-6_], respectively). This corresponds to faster elimination when compared to PEGylated liposomes of equivalent diameter (100 nm) and studied in the same zebrafish embryo model (≈15% left in circulation at the same 24 h timepoint).^[40]^ A direct comparison to PEG-based polymersomes in zebrafish embryos is not possible due to the lack of systematic circulation studies.^[59]^ However, evidence from mouse and rat studies have shown similar or better circulation times for PEG-based polymersomes compared to PEGylated liposomes.^[22,60]^ Our study represents a much required systematic circulation analysis of POx-based polymersomes, which highlights the need for further improvements in design to reach circulation times equivalent to PEG-based systems. We provide the design strategy of using copolymer blends.

## 3 Conclusion

We have presented a comprehensive study on POx-based polymersomes in terms of block length-dependent stability (DLS), protein fouling (FCS), hydration behavior (atomistic simulations), and their performance was cross-validated in vitro (macrophage association studies) and in vivo (zebrafish embryo study). These analyses demonstrate the benefit of maximizing the PMOXA part (at least *DP >* 6), while minimizing the PDMS block down to values that do not compromise vesicle stability (*DP* > 19). Fully atomistic simulations showed the importance of increasing PMOXA content to maximize interfacial water layer thickness and density for optimized antifouling behavior. While due to the computational limitations, the dynamics of the PDMS fraction/section could not yet be accurately modelled, together with an explicit incorporation and modulation of the dispersity it can be suggested as an interesting future avenue of research in polymer membranes and antifouling materials. Despite the need to maximize the hydrophilic block (PMOXA), it cannot be extended independently of the hydrophobic block (PDMS), since micelles are typically formed at a copolymer hydrophilic to total mass fraction of *f*_hydrophilic_ > 45%.^[50]^ We herein show that blending a long-chain PMOXA-based copolymer (non-vesicle-forming) with a vesicle-forming copolymer circumvents this limitation. These blends revealed reduced protein fouling, delayed macrophage association, and extended blood circulation times. The different antifouling mechanism of PMOXA versus PEG, which includes a lower mobility of PMOXA chains and reduced hydration of PMOXA at high density,^[33]^ highlight the need for independent optimization of POx-based versus PEG-based systems. Further improvements of POx-based polymersomes are required to reach a circulatory performance equivalent to PEG-based nanomedicines. Our study provides a systematic understanding of POx-based polymersomes in a biomedical context, critical to bringing this material system closer to clinical translation. Some possible next steps for further improving POx-based polymersomes are to test: i) further blends of copolymer lengths; ii) varied densities of overlapping PMOXA chains (achieved by modulating the mole fractions); iii) changing the polymer architecture to a cyclic version; and iv) using more flexible POx polymers such as PMeOzi. Overall, our study provides a much needed, systematic understanding of PMOXA-polymersomes in a biomedical context which is critical for moving polymersomes closer to clinical translation for potential biomedical applications in the fields of sensing, therapy, immunotherapy, and vaccination.

## 4 Experimental Section

All experimental details are shown in the Supporting Information. All experiments were approved by the Norwegian authorities regulating animal research.

## Supplementary Material

Supplementary Materials

## Figures and Tables

**Table 1:** Summary of all the experimentally used and simulated PMOXA-*b*-PDMS-*b*-PMOXA copolymers, including *DPs* and hydrophilic fractions (*f*_hydrophilic_ = *M_w_* hydrophilic blocks/*M_w_* copolymer x 100). Names represent *DP* of corresponding blocks (e.g., PMOXA_6_-*b*-PDMS_65_-*b*-PMOXA_6_ is denoted as 6-65-6).

Experimental copolymers	*f*_hydrophilic_ [%]	Simulated copolymers	Short names	*f*_hydrophilic_ [%]
21-65-21^a)^	43	6-37-6	6	30
12-63-12^b)^	32	5-31-5	5	30
10-87-10^d)^	22	4-25-4	4	31
7-42-7^e)^	30	3-37-3	–	20
6-65-6^c)^	20	3-19-3	3	32
6-34-6^b)^	32	2-13-2	2	37
3-19-3^b)^	32	1-7-1	1	37

a) Used in blends as “long” copolymer

b) From Ref. [47]

c) Used in blends as “short” copolymer

d) From Ref. [48]

e) From Ref. [49].

## Data Availability

The data that support the findings of this study are available from the corresponding author upon reasonable request.

## References

[1] Discher BM, Won Y-Y, Ege DS, Lee JCM, Bates FS, Discher DE, Hammer DA (1999). Science.

[2] Iqbal S, Blenner M, Alexander-Bryant A, Larsen J (2020). Biomacromolecules.

[3] Matoori S, Leroux JC (2020). Mater Horiz.

[4] Zartner L, Muthwill MS, Dinu IA, Schoenenberger CA, Palivan CG (2020). J Mater Chem B.

[5] Araste F, Aliabadi A, Abnous K, Taghdisi SM, Ramezani M, Alibolandi M (2021). J Controlled Release.

[6] Oerlemans RAJF, Timmermans SBPE, van Hest JCM (2021). ChemBioChem.

[7] Scheerstra JF, Wauters AC, Tel J, Abdelmohsen LKEA, van Hest JCM (2022). Mater Today Adv.

[8] Stater EP, Sonay AY, Hart C, Grimm J (2021). Nat Nanotechnol.

[9] Bilardo R, Traldi F, Vdovchenko A, Resmini M (2022). Wiley Inter-discip Rev: Nanomed Nanobiotechnol.

[10] Shan X, Liu C, Yuan Y, Xu F, Tao X, Sheng Y, Zhou H (2009). Colloids Surf, B.

[11] Perry JL, Reuter KG, Kai MP, Herlihy KP, Jones SWJ, Luft C, Napier M, Bear JE, Desimone JM (2012). Nano Lett.

[12] Aggarwal P, Hall JB, McLeland CB, Dobrovolskaia MA, McNeil SE (2009). Adv Drug Delivery Rev.

[13] Wani TU, Raza SN, Khan NA (2020). Polym Bull.

[14] Schöttler S, Becker G, Winzen S, Steinbach T, Mohr K, Landfester K, Mailänder V, Wurm FR (2016). Nat Nanotechnol.

[15] Qiao R, Fu C, Li Y, Qi X, Ni D, Nandakumar A, Siddiqui G, Wang H, Zhang Z, Wu T, Zhong J (2020). Adv Sci.

[16] Prozeller D, Pereira J, Simon J, Mailänder V, Morsbach S, Landfester K (2019). Adv Sci.

[17] Alberg I, Kramer S, Schinnerer M, Hu Q, Seidl C, Leps C, Drude N, Möckel D, Rijcken C, Lammers T, Diken M (2020). Small.

[18] Friedl JD, Nele V, De Rosa G, Bernkop-Schnürch A (2021). Adv Funct Mater.

[19] Du H, De Oliveira FA, Albuquerque LJC, Tresset G, Pavlova E, Huin C, Guégan P, Giacomelli FC (2020). Langmuir.

[20] de Oliveira FA, Albuquerque LJC, Riske KA, Jäger E, Giacomelli FC (2020). Colloid J Interface Sci.

[21] de Oliveira FA, Albuquerque LJC, Castro CE, Riske KAI, Bellettini C, Giacomelli FC (2022). Colloids Surf, B.

[22] Photos PJ, Bacakova L, Discher B, Bates FS, Discher DE (2003). J Controlled Release.

[23] Li M, Jiang S, Simon J, Paßlick D, Frey ML, Wagner M, Mailander V, Crespy D, Landfester K (2021). Nano Lett.

[24] Richtering W, Alberg I, Zentel R (2020). Small.

[25] Bigini P, Gobbi M, Bonati M, Clavenna A, Zucchetti M, Garattini S, Pasut G (2021). Nat Nanotechnol.

[26] McSweeney MD, Shen L, DeWalle AC, Joiner JB, Ciociola EC, Raghuwanshi D, Macauley MS, Lai SK (2021). J Controlled Release.

[27] Sedlacek O, Hoogenboom R (2020). Adv Ther.

[28] Thi TTH, Pilkington EH, Nguyen DH, Lee JS, Park KD, Truong NP (2020). Polymers.

[29] Trachsel L, Romio M, Ramakrishna SN, Benetti EM (2020). Adv Mater Interfaces.

[30] Zahoranová A, Luxenhofer R (2021). Adv Healthcare Mater.

[31] Poon W, Kingston BR, Ouyang B, Ngo W, Chan WCW (2020). Nat Nanotechnol.

[32] Penna M, Yarovsky I (2020). Nanoscale.

[33] Penna M, Ley KJ, Belessiotis-Richards A, Maclaughlin S, Winkler DA, Yarovsky I (2019). J Phys Chem C.

[34] Le TC, Penna M, Winkler DA, Yarovsky I (2019). Sci Rep.

[35] Molino PJ, Yang D, Penna M, Miyazawa K, Knowles BR, MacLaughlin S, Fukuma T, Yarovsky I, Higgins MJ (2018). ACS Nano.

[36] Penna M, Ley K, MacLaughlin S, Yarovsky I (2016). Faraday Discuss.

[37] Zalipsky S, Hansen CB, Oaks JM, Allen TM (1996). J Pharm Sci.

[38] Finnegan JR, Pilkington EH, Alt K, Rahim MA, Kent SJT, Davis P, Kempe K (2021). Chem Sci.

[39] Liu K, Wang X, Li-Blatter X, Wolf M, Hunziker P (2020). ACS Appl Bio Mater.

[40] Dal NJK, Kocere A, Wohlmann J, Van Herck S, Bauer TA, Resseguier J, Bagherifam S, Hyldmo H, Barz M, De Geest BG, Fenaroli F (2020). Small.

[41] Sieber S, Grossen P, Detampel P, Siegfried S, Witzigmann D, Huwyler J (2017). J Controlled Release.

[42] Sieber S, Grossen P, Bussmann J, Campbell F, Kros A, Witzigmann D, Huwyler J (2019). Adv Drug Delivery Rev.

[43] Cörek E, Rodgers G, Siegrist S, Einfalt T, Detampel P, Schlepütz CM, Sieber S, Fluder P, Schulz G, Unterweger H, Alexiou C (2020). Small.

[44] Tao J, Wei Z, Xu M, Xi L, Cheng Y, Lee SM, Ge W, Zheng Y (2021). Small.

[45] Einfalt T, Witzigmann D, Edlinger C, Sieber S, Goers R, Najer A, Spulber M, Onaca-Fischer O, Huwyler J, Palivan CG (2018). Nat Commun.

[46] Porta F, Ehrsam D, Lengerke C, Meyer Zu Schwabedissen HE (2018). Mol Pharmaceutics.

[47] Itel F, Chami M, Najer A, Lörcher S, Wu D, Dinu IA, Meier W (2014). Macromolecules.

[48] Egli S (2011). PhD Thesis.

[49] Lörcher S, Meier W (2017). Eur Polym J.

[50] Discher DE, Eisenberg A (2002). Science.

[51] Penders J, Pence IJ, Horgan CC, Bergholt MS, Wood CS, Najer A, Kauscher U, Nagelkerke A, Stevens MM (2018). Nat Commun.

[52] Cai D, Neyer A, Kuckuk R, Heise HM (2010). J Mol Struct.

[53] Kari OK, Ndika J, Parkkila P, Louna A, Lajunen T, Puustinen A, Viitala T, Alenius H, Urtti A (2020). Nanoscale.

[54] Rigler R, Mets Ü, Widengren J, Kask P (1993). Eur Biophys J.

[55] Rigler P, Meier W (2006). J Am Chem Soc.

[56] Martinez-Moro M, Di Silvio D, Moya SE (2019). Biophys Chem.

[57] Najer A, Blight J, Ducker CB, Gasbarri M, Brown JC, Che J, Høgset H, Saunders C, Ojansivu M, Lu Z, Lin Y (2022). ACS Cent Sci.

[58] Tcherniak A, Reznik C, Link S, Landes CF (2009). Anal Chem.

[59] Kocere A, Resseguier J, Wohlmann J, Skjeldal FM, Khan S, Speth M, Dal NJK, Ng MYW, Alonso-Rodriguez N, Scarpa E, Rizzello L, Battaglia G (2020). eBioMedicine.

[60] Lee JS, Ankone M, Pieters E, Schiffelers RM, Hennink WE, Feijen J (2011). Controlled J Release.

