# Supplementary material for: Block Length-Dependent Protein Fouling on Poly(2-oxazoline)-Based Polymersomes: Influence on Macrophage Association and Circulation Behavior
Source: Small. Author manuscript; Available in PMC 2024 Jan 6. (PMC7615485; doi:10.1002/smll.202201993)
Supplement: Supplementary Materials [file EMS175899-supplement-Supplementary_Materials.pdf]

NANO MICRO small

Supporting Information

for *Small,* DOI: 10.1002/smll.202201993

Block Length-Dependent Protein Fouling on Poly(2-oxazoline)-Based Polymersomes: Influence on Macrophage Association and Circulation Behavior

Adrian Najer, Alexis Belessiotis-Richards, Hyemin Kim, Catherine Saunders, Federico Fenaroli, Christopher Adrianus, Junyi Che, Renée L. Tonkin, Håkon Høgset, Samuel Lörcher, Matthew Penna, Stuart G. Higgins, Wolfgang Meier, Irene Yarovsky,* and Molly M. Stevens**

Supporting Information

Block Length-Dependent Protein Fouling on Poly(2-oxazoline)-Based Polymersomes: Influence on Macrophage Association and Circulation Behavior

Adrian Najer,‡, Alexis Belessiotis-Richards,‡ Hyemin Kim, Catherine Saunders, Federico Fenaroli, Christopher Adrianus, Junyi Che, Renée L. Tonkin, Håkon Høgset, Samuel Lörcher, Matthew Penna, Stuart G. Higgins, Wolfgang Meier, Irene Yarovsky* and Molly M. Stevens**

Materials and Methods

All the data was plotted using GraphPad Prism 9.0.0.

Polymersome Formation

PMOXA-*b*-PDMS-*b*-PMOXA block copolymers from our previous studies[1–3] and two commercial block copolymers (Polymer Source, P18140D-MOXZDMSMOXZ (6-65-6), Mw: 500-4800-500 g/mol, *Đ* = 1.3 and P18140A-MOXZDMSMOXZ (21-65-21), Mw: 1800-4800-1800 g/mol, *Đ* = 1.35) were used. First, the copolymers were dissolved in ethanol at 20 mg/mL using a bath sonicator. The desired amount was transferred to glass vials, and ethanol evaporated by hand using a nitrogen stream until dryness. The thin film was then hydrated in phosphate buffered saline (PBS, Sigma-Aldrich, D8537) and stirred for at least overnight at RT. The solution was then extruded through 0.1 µm polycarbonate membranes at least 15 times (Avanti, Sigma-Aldrich, 610000). Subsequent purification was performed using PD Minitrap or PD MidiTrap columns (GE Healthcare) equilibrated in PBS. SRB loading was performed by hydration of the thin film with 1 mM SRB in PBS and following the same steps as above but using two columns sequentially to purify away all the free dye. Encapsulation was quantified by measuring fluorescence of the purified samples in a plate reader (SpectraMax M5, Molecular Devices). To label the membrane of polymersomes, 1,1'-Dioctadecyl-3,3,3',3'-Tetramethylindodicarbocyanine Perchlorate (0.5 mol%, DiD, Thermo Fisher Scientific, D307) was added to the copolymer ethanol stocks before forming the films. After running through a PD MidiTrap column equilibrated in PBS, the fluorescence of the polymersomes was measured on a plate reader (SpectraMax M5, Molecular Devices), which also allowed adjusting all the samples to the same amount of fluorescence for cell uptake studies (see below). Samples were sterilized by means of filtration through a 0.22 µm syringe filter (Millipore, SLGV013SL) inside a biosafety cabinet and subsequently stored at 4 °C.

Giant polymersomes at the microscale were formed by the spontaneous hydration method.[4] Briefly, a 7 mL glass vial was first plasma treated for 1 min, then a small volume (40 µL) of

copolymer stock solution in ethanol at 6 mg/mL was added and dispersed on the vial bottom and the ethanol was allowed to evaporate slowly. Once dried, the film was further dried in a vacuum desiccator for 5 h. Then the film was hydrated with 0.6 mL 0.3 M sucrose and the vial sealed with parafilm and incubated without agitation at 60 °C overnight. The next day the giant polymersomes were pipetted away from the glass vial bottom and used unpurified.

DLS and Zeta Potential Measurements

Measurements ($n = 3$) were conducted on a Malvern Zetasizer Nano-ZS. 70 µL of polymersome solution in PBS was pipetted into single use microcuvettes for DLS. For zeta potential measurements, 950 µL of 0.3 M sucrose was mixed with 50 µL polymersome samples in PBS and transferred to zeta potential cuvettes.

TEM and Cryo-TEM

After plasma cleaning TEM grids (Electron Microscopy Sciences, CF200-Cu, 215-412-8400) for 1 min, 5 µL of polymersome solution in PBS (0.5 mg/mL) was added, incubated for 1 min and blotted away. Sample addition was followed by two ddH$_2$O drop washes before negatively staining the sample with two drops of 2 wt% uranyl acetate in water (0.45 µm filtered), whilst leaving the second drop on the grid for 15 s before blotting away. The overnight dried grids were then imaged on a JEOL 2100F.

For cryo-TEM, a glow-discharged Quantifoil R2/2 grid (400 copper mesh, carbon coated, Quantifoil Micro Tools GmbH, Großlöbichau, Germany) was loaded with 3 µL of polymersome solution (8 mg/mL) in PBS and incubated for 10 s, blotted away for 4 s and plunged into liquid ethane using an automatic plunge freezer FEI Vitrobot (Thermo Fisher Scientific, Waltham, MA, USA) operated at 21 °C and a 100% relative humidity. The samples were then imaged on a JEOL JEM-2100f (JEOL Ltd., Tokyo, Japan) equipped with a TVIPS TemCam-XF416 CMOS camera (Tietz Video and Image Processing Systems GmbH, Gauting, Germany).

FBS and Clusterin Labeling

Fetal bovine serum (FBS) and clusterin were randomly labeled via free amine coupling using NHS-activated dye. Briefly, an aliquot of 50 µL FBS (Gibco, 10500-054, lot 08F2381K) was added to 0.2 mL carbonate buffer (0.1 M carbonate, Sigma-Aldrich, 0.15 M NaCl, VWR, pH 8.0). Then, 2.5 µL DMSO containing 0.67 mg of OG488-NHS (Invitrogen, 06149 Oregon Green™ 488 Carboxylic Acid, Succinimidyl Ester, 6-isomer) was mixed in and the reaction was incubated under shaking at room temperature for 3 h. For clusterin, 25 µL of human

clusterin (2bscientific, CLU-H5227) at 2 mg/mL (50 μg) in phosphate buffer (0.1 M, 0.05 M NaCl, pH 7.4) was mixed with 0.25 μL 100 mg/mL OG488-NHS (25 μg) in DMSO followed by incubation under shaking at room temperature for 3 h. Free dye was removed from the labeled FBS components (FBS-OG) and clusterin-OG by sequential SEC using one PD MiniTrap (GE Healthcare) and one PD MidiTrap (GE Healthcare) equilibrated in PBS. This final FBS-OG stock (is now a 1/30 dilution of FBS in PBS) and clusterin-OG stock (33 μg/mL) were then sterile filtered (0.2 μm), aliquoted and stored at -20 °C.

Confocal Fluorescence Imaging

First 280 μL of a 1/30 dilution of the FBS-OG488 stock in PBS (finally a 1/900 dilution of FBS) was transferred to each well of an 8-well ibidi plate (80827, ibidi, Germany). Then, 20 μL of the giant polymersome stock in 0.3 M sucrose was added and the plate was incubated static at 37 °C for 4 h. Then, 20 μL of 100 μM Bodipy630 (Thermo Fisher Scientific, D10000, NHS ester deactivated by overnight incubation in PBS) in PBS was added to visualize the membrane before imaging the samples using a commercial LSM 880 (Carl Zeiss, Jena, Germany). Three random spots were chosen per sample for imaging and all the imaging conditions were kept the same for all samples. The Bodipy630 red channel and phase channel images were brightness adjusted in Fiji to optimize visualization of giant location, while the FBS-OG488 channel images were left unmodified.

FCS Measurements

Polymersome samples in PBS (4 mg/mL) were first diluted 1/10 in a 1/20 dilution of the FBS-OG stock in PBS (finally 1/600 FBS dilution) and incubated at 37 °C, 450 RPM using a ThermoMixer. For the clusterin binding experiment, polymersome samples in PBS (4 mg/mL) were mixed 1 to 1 with clusterin-OG stock (see above, 33 μg/mL in PBS) and incubated at 37 °C for 7 h, 450 RPM using a ThermoMixer. Aliquots of 5 μL were taken to conduct FCS measurements on a commercial LSM 880 (Carl Zeiss, Jena, Germany) equilibrated at 37 °C, with data analysis performed with PyCorrfit program 1.1.6.[5] A dilution series of free OG488 in PBS (D = 5.49 x 10^{-6} cm^2/s at 37 °C, D = 4.1 x 10^{-6} cm^2/s at 25 °C)[6] was employed to calibrate and yield the x-y dimension of the confocal volume (ω_{xy}^2), which is necessary in order to calculate diffusion coefficients (D) for all the samples and subsequent transformation to hydrodynamic diameters using the Stokes-Einstein equation.

The sample droplet was deposited in an ibidi 8-well plate (80827, ibidi, Germany) and excited with a 488 nm Ar^+ laser focused 200 μm above the glass plate. Fluorescence intensity

fluctuations were recorded through a 40x C-Apochromat water immersion objective (NA 1.2) and applying appropriate filter sets. 25 intensity traces of 5 s each were recorded and autocorrelated per sample, whilst the shown auto-correlation curves represent the average curves of the whole measurement of 125 s. FBS-OG or clusterin-OG data was first fitted with one component fits $G_{1comp}(\tau)$ and all the data was subsequently analyzed with two-component fits $G_{2comp}(\tau)$, fixing the diffusion time τ_D of the first component to the obtained free FBS-OG or clusterin-OG diffusion time and the second component to a diffusion time corresponding to 100 nm diameter nanoparticles (τ_1, τ_2). For 3-19-3, the second component (τ_2) was not fixed, due to aggregation. A triplet fraction T with corresponding triplet time τ_{trip} was included and fixed between 1 – 10 µs and the structural parameter SP, which is the ratio of height to width of the confocal volume was set to 5. The fits yielded the corresponding fractions (F_1, F_2; $N = n1 + n2$) with F_2 *100 being plotted as particle fraction. All the data points (*n* = 25) for all the three independent experiments (*N* = 3) are shown in Figure S6 and S7, while the medians for the three independent experiments are summarized in Figure 2c,d.

$$G_{1comp}(\tau) = \left(1 + \frac{T}{1-T} e^{\frac{-\tau}{\tau_{trip}}}\right) * \frac{1}{N * \left(1 + \frac{\tau}{\tau_D}\right) * \sqrt{1 + \frac{\tau}{SP^2 \tau_D}}}$$

$$G_{2comp}(\tau) = \left(1 + \frac{T}{1-T} e^{\frac{-\tau}{\tau_{trip}}}\right) * \frac{1}{N} * \left[\frac{F_1}{\left(1 + \frac{\tau}{\tau_1}\right) * \sqrt{1 + \frac{\tau}{SP^2 \tau_1}}} + \frac{1 - F_1}{\left(1 + \frac{\tau}{\tau_2}\right) * \sqrt{1 + \frac{\tau}{SP^2 \tau_2}}}\right]$$

SPARTA®

SPARTA® has been described elsewhere.[7] We herein used a custom confocal Raman microspectroscope assembled on a Cerna platform (Thorlabs, UK). The components included a spectrograph (HoloSpec-F/1.8-NIR, Andor, UK) and an iDus 416ALDC-DD (Andor, UK) thermoelectrically cooled (−60 °C) backilluminated CCD camera. The 63×/1.0 NA WI objective (W Plan-Aprochromat, Zeiss, Oberkochen, Germany) was directly placed into the polymersome PBS solution and a Raman excitation laser at 785 nm (200 mW, Cheetah, Sacher Laser Technik, Germany) was employed. Many excitation wavelengths can be used for Raman scattering. While shorter wavelength excitation offers increased Raman signal, here we selected a longer wavelength to reduce potential laser burning of the sample, or sample photoluminescence. Each trapped particle was measured for 20 s, before disabling the laser for 1 s to release the trapped polymersome and allow a new polymersome to diffuse into the trap. Blank PBS measurements were used for background subtraction. Custom MATLAB scripts for

cosmic spike removal, spectral response correction (785 nm reference standard, National Institute of Standards and Technology, US), background subtraction, baseline correction, smoothing, and normalization were employed for Raman spectra analysis. PCA analysis was performed using the PLS ToolBox Version 8.8.1 (Eigenvector Research, Inc. WA, USA).

Simulations Details

All-atom classical molecular dynamics (MD) simulations were performed using the graphical processing unit (GPU) accelerated NAMD (version 2.13) software[8] with the CGENFF36 force-field.[9,10] Charges and atom types for the POx ligands were obtained via analogy and additional bond, angle and dihedral parameters for the attachment ligands to the surface and within the POx ligands were generated using the ffTK tool in VMD.[11] All simulations were conducted in the isothermal–isobaric ensemble (NPT) at 310K using a Langevin thermostat with a damping coefficient of 1 ps^{-1}; whereas the Nosé-Hoover Langevin piston pressure control (with piston oscillation period of 0.2 ps and piston decay time of 0.1 ps) provided a constant atmospheric pressure environment.[12,13] Long range electrostatic interactions were calculated by the particle-mesh Ewald method with a grid spacing of 1.0 Å. A cut-off radius of 12 Å was used for the short range (i.e. Lennard-Jones) interaction with a switching implemented at 10 Å. Each system was run for 50 ns of production MD for analyses after 50 ns of equilibration.

Membrane construction: In order to construct membranes from the PMOXA-*b*-PDMS-*b*-PMOXA triblock polymers, the chains were aligned parallel in a square formation. We first restrained the x-y motion of the nitrogen atoms within the PMOXA monomers as well as the terminal silicon atoms in the PDMS. We then slowly reduced the z-position of each group, without pressure coupling, in order to compress the membrane to a realistic thickness from the experimental thicknesses taken from Itel et al.[1] Once compressed, we ran NPT equilibration on the system for 50 ns using the same parameters as above.

Cell Interaction Study by Imaging and Flow Cytometry

RAW 264.7 cell line was cultured in DMEM medium (high glucose) containing 10% (v/v) FBS, P/S (1% (v/v), Sigma-Aldrich) and L-glutamine (1% (v/v), Sigma-Aldrich, G7513, now called full medium). Briefly, 1 mL 300 000 RAW 264.7 cells/mL per well were seeded into 24 well-plates (for flow cytometry) or 0.8 mL 300 000 RAW 264.7 cells/mL in 4 well ibidi plates (ibidi, Germany, for imaging). After incubation for 24 h at 37 °C, the spent medium was replaced with fresh full medium and polymersome solution in PBS using PBS/media ratios of 1/4 or 1/10. All

polymersome samples were first diluted to yield the same fluorescence in PBS (DiD, measured on plate reader), in order to correct for difference in sample formation efficacy (e.g., loss during extrusion) and normalize to the final signal producing property (DiD fluorescence). After incubation for another 2 – 4 h at 37 °C, the cells were washed three times with PBS, transferred to Eppendorf tubes (for flow cytometry samples), fixed for at least 15 min using 4% (v/v) PFA in PBS, washed twice with PBS and transferred to flow cytometry tubes. Flow cytometry was conducted on a BD LSRFortessa I, keeping all the settings constant for all the measurements. Median fluorescence values were exported and % of median fluorescence was calculated by setting 6-65-6 samples to 100%. For fluorescence imaging, the samples were further stained for 15 min using 1/500 WGA-488 and 1/1000 DAPI in PBS and washed again for 3 times with PBS. A Nikon Ti Microscope with a 100x oil immersion objective was used to obtain overview images which were taken randomly, whilst z-stacks were recorded of cells that had a high number of particles inside to visualize intracellular location. All the imaging settings were kept constant for the particle red channel, whilst the other channels were adjusted independently. EpiDEMIC plugin in Icy (50 iterations) was employed to deconvolve the z-stacks.[14] Images were processed in Fiji.

Zebrafish Embryo Injections and Imaging Analysis

Following fertilization, wild-type zebrafish embryos were kept in petri dishes at 28.5 °C in zebrafish egg water supplemented with phenylthiourea (0.003% (w/v)). On day two post fertilization, zebrafish embryos were sedated in egg water containing tricaine (0.02% (w/v)) and placed on a dish filled with a hardened solution of 2% (w/v) agarose. Nanoparticle solutions were loaded onto borosilicate glass needles previously prepared using a pipette puller (P-97, Sutter instruments). The needles, connected to an Eppendorf Femtoject Express microinjector, were then placed on a micromanipulator (Narishige MN-153). Injections were all performed in the posterior cardinal vein with a volume of 5 nanoliters. For both polymersome samples, at 2 min, 1 h, 4 h, 8 h and 24 h images at a magnification of 30X and 120X were acquired using a stereomicroscope (DFC365FX, 1.0X planapo lens). Analysis of the nanoparticle circulation over time has been analyzed as described elsewhere.[15] Briefly, using the program ImageJ, the fluorescence of the artery (at magnification 120X) was estimated for each time point and normalized by the overall fluorescence of the zebrafish (at a magnification of 30X). After removing the background signal, the loss of fluorescence intensity over time (at the 2 min time point nanoparticles were all considered in circulation) was plotted. All experiments were approved by the Norwegian authorities regulating animal research.

Statistical Analysis

The specific sample sizes and statistical tests and post hoc analysis are specified in the figure captions. Data normalization and representation is also explained in each caption. All the data was plotted and analyzed using GraphPad Prism 9.0.0.

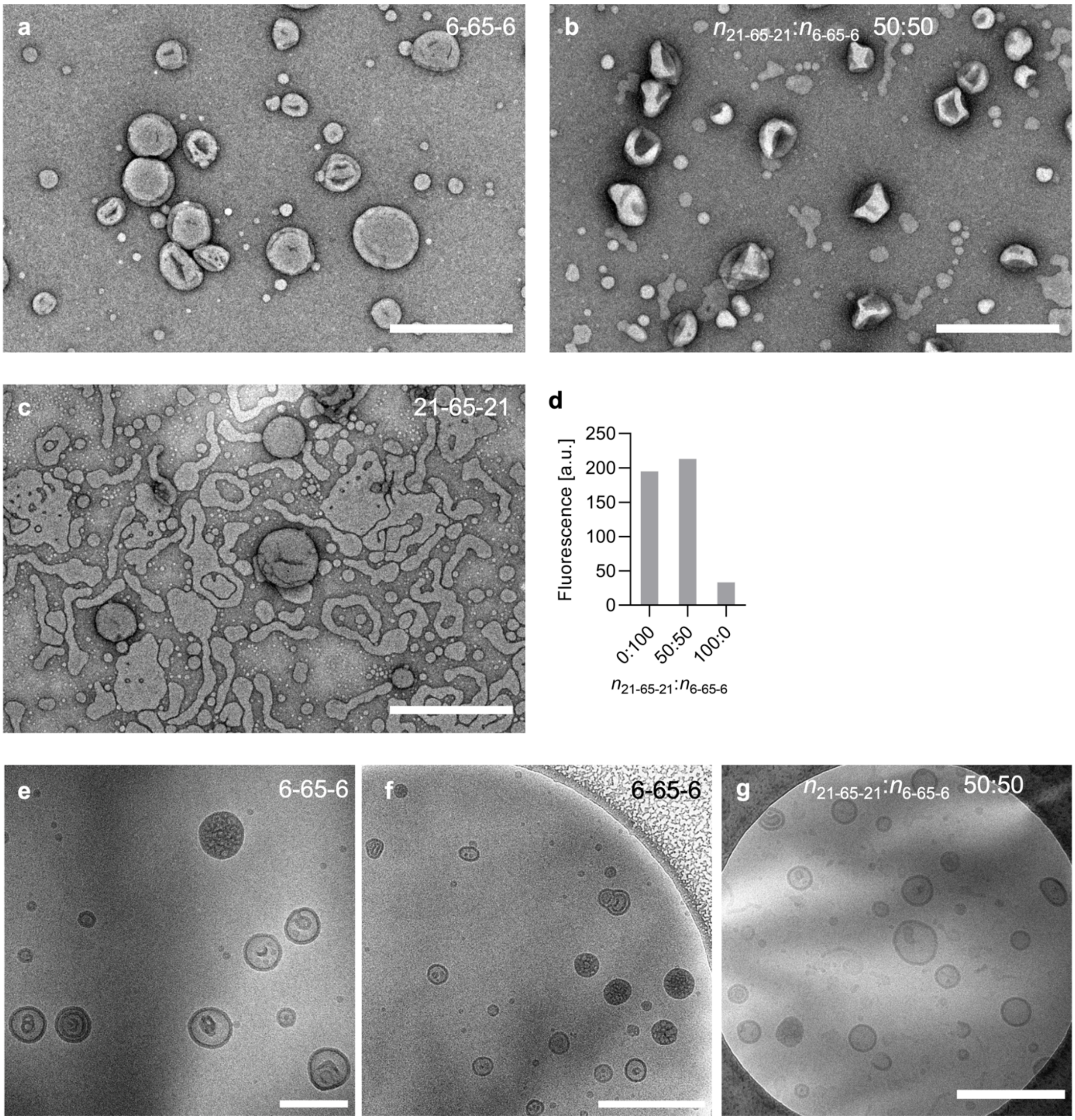

Figure S1. Polymersome characterization. (a) TEM image of polymersomes made from 6-65-6 copolymer. Scale bar, 500 nm. (b) TEM of a 50 mol% blend of 6-65-6 and 21-65-21. (c) TEM image of assemblies made from 21-65-21 copolymer. Scale bar, 500 nm. (d) Fluorescence of nanoassemblies made from 6-65-6, 50% blend, or 21-65-21 copolymers after loading with SRB and SEC purification (n = 1). (e,f) Cryo-TEM images of (a). Scale bars, 200 nm (e) and 500 nm (f). (g) Cryo-TEM image of (b). Scale bar, 500 nm.

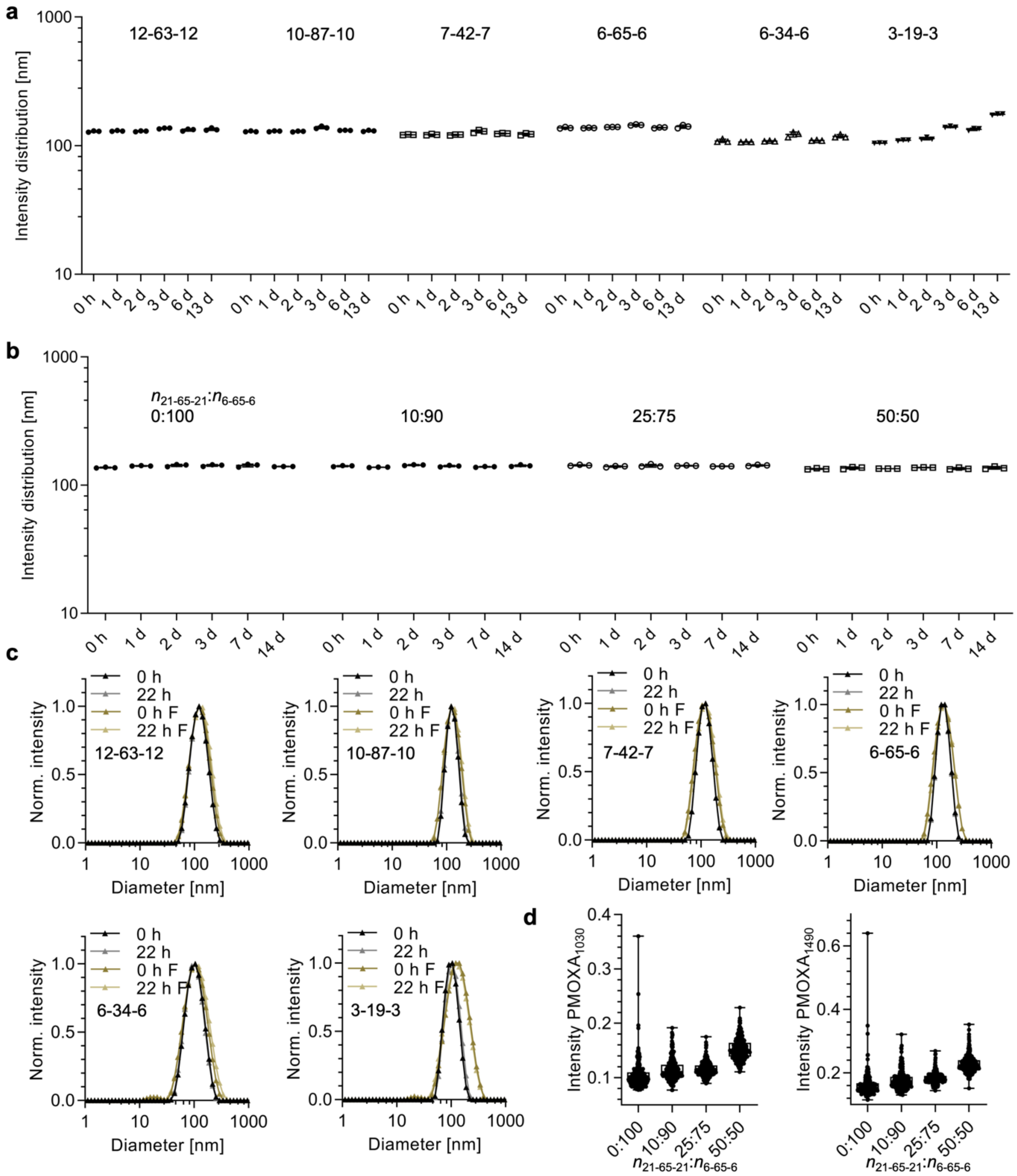

Figure S2. Polymersome stability. (a,b) DLS peak values (intensity distribution) for various polymersomes incubated at 37 °C in PBS (mean ± s.d. of technical triplicates). (c) DLS measurement (intensity distributions) of assemblies made from various copolymers (mean of technical triplicates) after incubation in PBS or 10 v/v% FBS (F) at 37 °C, after mixing and 22 h later. (d) SPARTA® data (from Figure 1h) showing Raman peak intensity at 1030 and 1490 cm^{-1} Raman shift for all the traps (n > 200 traps each). All data was normalized to the main PDMS peak (Si-C at 708 cm^{-1}) to compare the variations in PMOXA content.

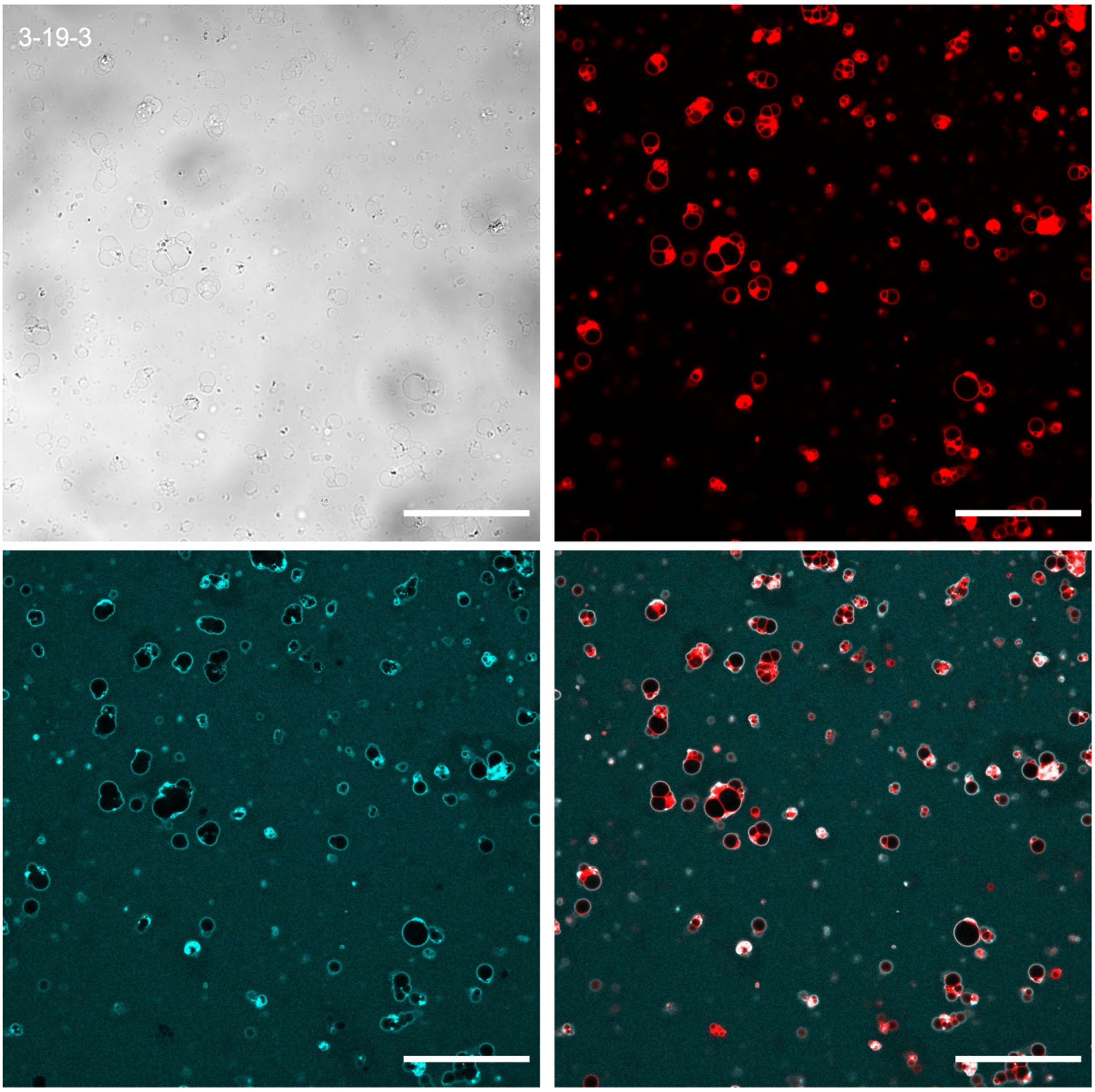

Figure S3. Imaging of protein fouling on giant polymersomes. Binding of randomly labeled fetal bovine serum (FBS-OG488) to giant microscaled polymersomes made from copolymer 3-19-3, after 4 h incubation at 37 °C. Red channel shows membrane stain and cyan is FBS-OG488. Scale bars, 50 µm. (Figure 2b shows a zoomed region of this image).

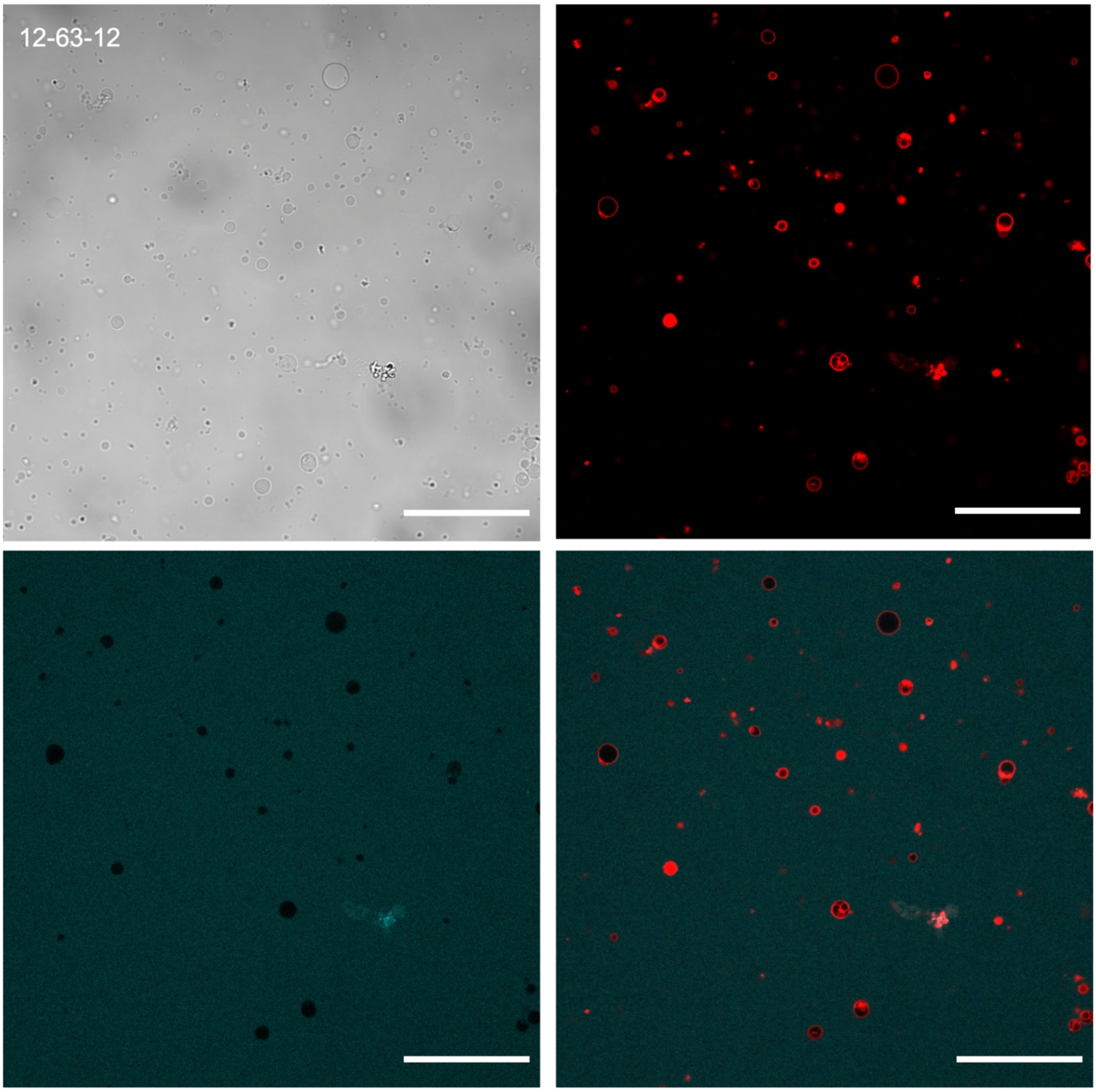

Figure S4. Imaging of protein fouling on giant polymersomes. Binding of randomly labeled fetal bovine serum (FBS-OG488) to giant micron scaled polymersomes made from copolymer 12-63-12, after 4 h incubation at 37 °C. Red channel shows membrane stain and cyan is FBS-OG488. Scale bars, 50 µm.

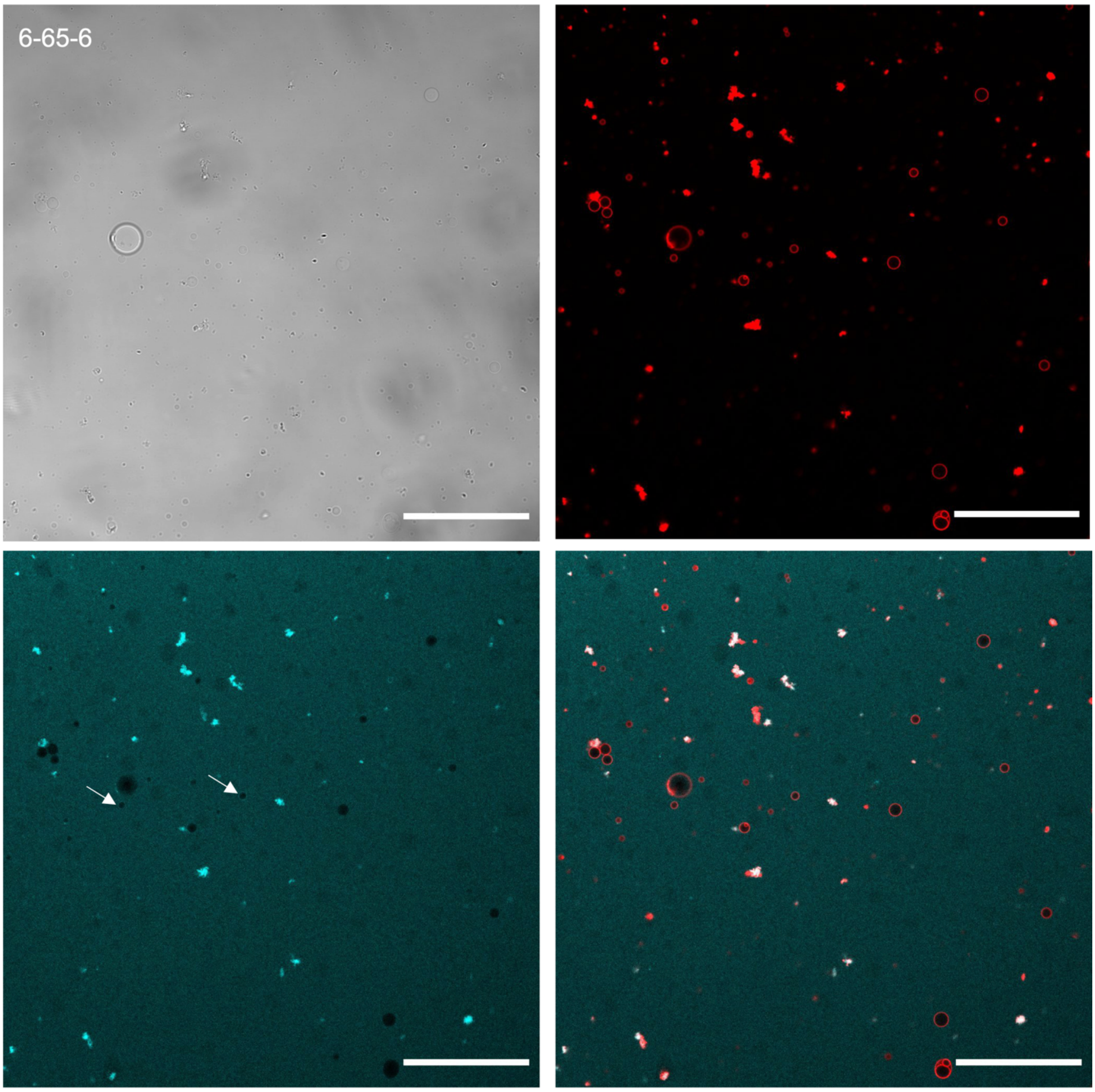

Figure S5. Imaging of protein fouling on giant polymersomes. Binding of randomly labeled fetal bovine serum (FBS-OG488) to giant micron scaled polymersomes made from copolymer 6-65-6, after 4 h incubation at 37 °C. Red channel shows membrane stain and cyan is FBS-OG488. White arrows indicate some FBS-OG488 binding to small vesicles. Scale bars, 50 µm.

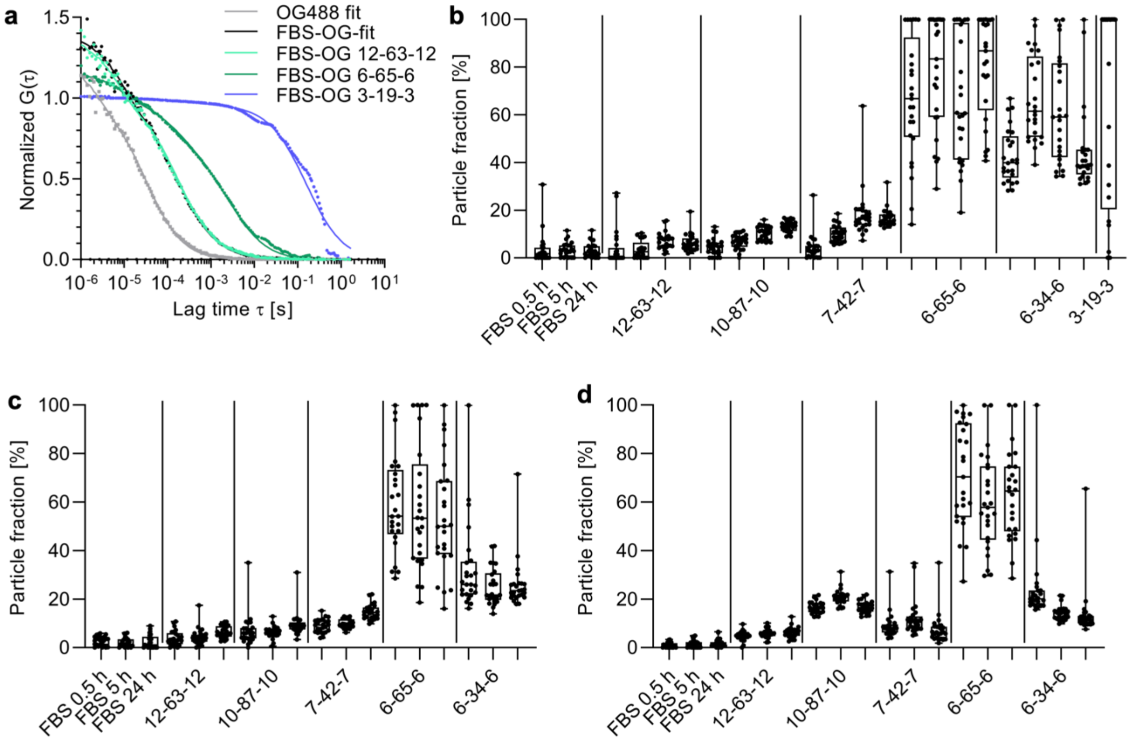

Figure S6. FCS analysis of protein fouling on polymersomes. Normalized average autocorrelation curves from FCS measurements with FBS-OG488 mixed with unlabeled polymersomes (two-component fits) and controls of free FBS-OG488 and OG488 dye alone. (b-d) 3 independent experimental repeats of particle fraction for two-component fits of FCS curves (as shown in (a)) revealing extent of protein fouling on polymersome surface (n = 25 technical repeats each). Box-plots: Center line, the median; box limits, upper and lower quartiles; whiskers, minimum and maximum values. Median values from these 3 independent experiments are summarized in Figure 2c.

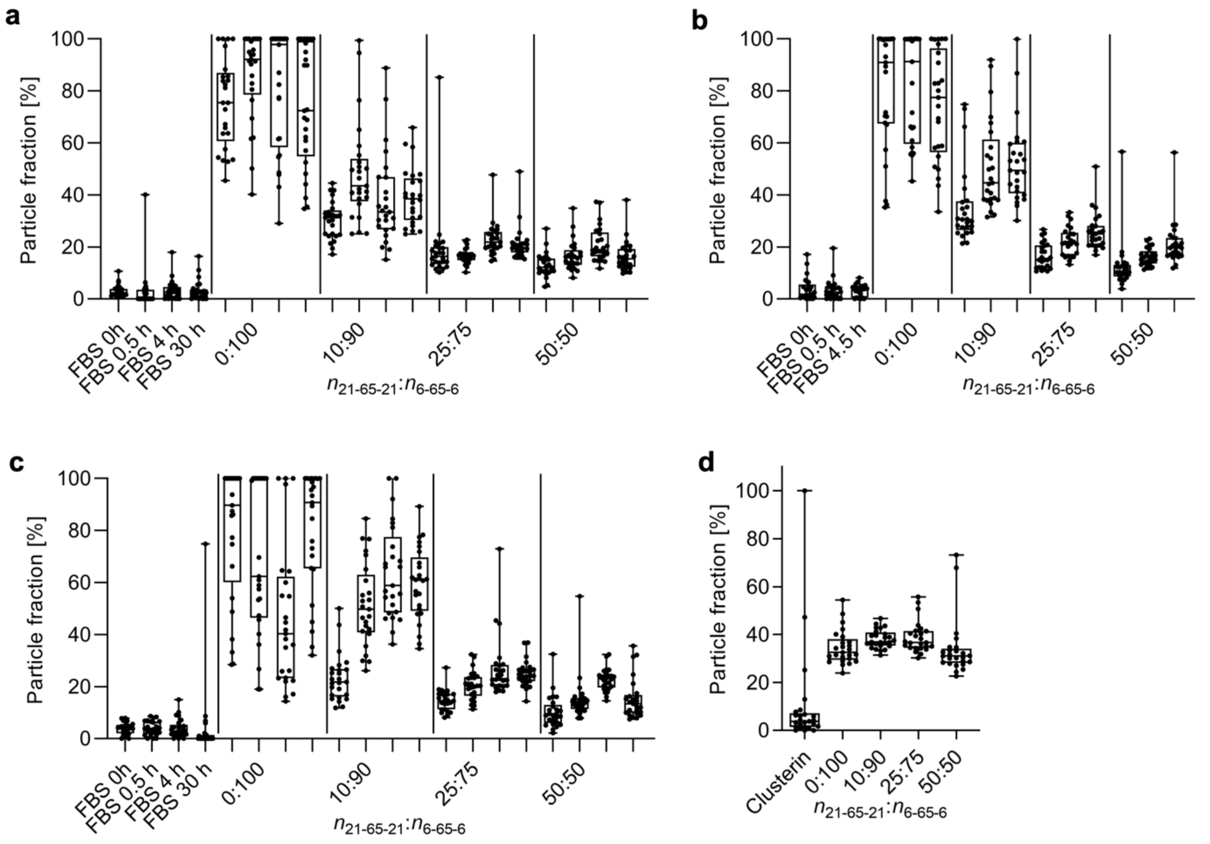

Figure S7. FCS analysis of protein fouling and clusterin binding on polymersomes made from copolymer blends of 6-65-6 and 21-65-21. (a-c) 3 independent experimental repeats of particle fraction for two-component fits of FCS curves (as shown in Figure S6a) revealing extent of protein fouling on polymersome surface (*n* = 25 technical repeats each). (d) Particle fraction for two-component fits of FCS curves revealing the extent of clusterin-OG488 binding on polymersome surfaces after incubation for 7 h (*n* = 25 technical repeats each). Box-plots: Center line, the median; box limits, upper and lower quartiles; whiskers, minimum and maximum values. Median values from these 3 independent experiments are summarized in Figure 2d.

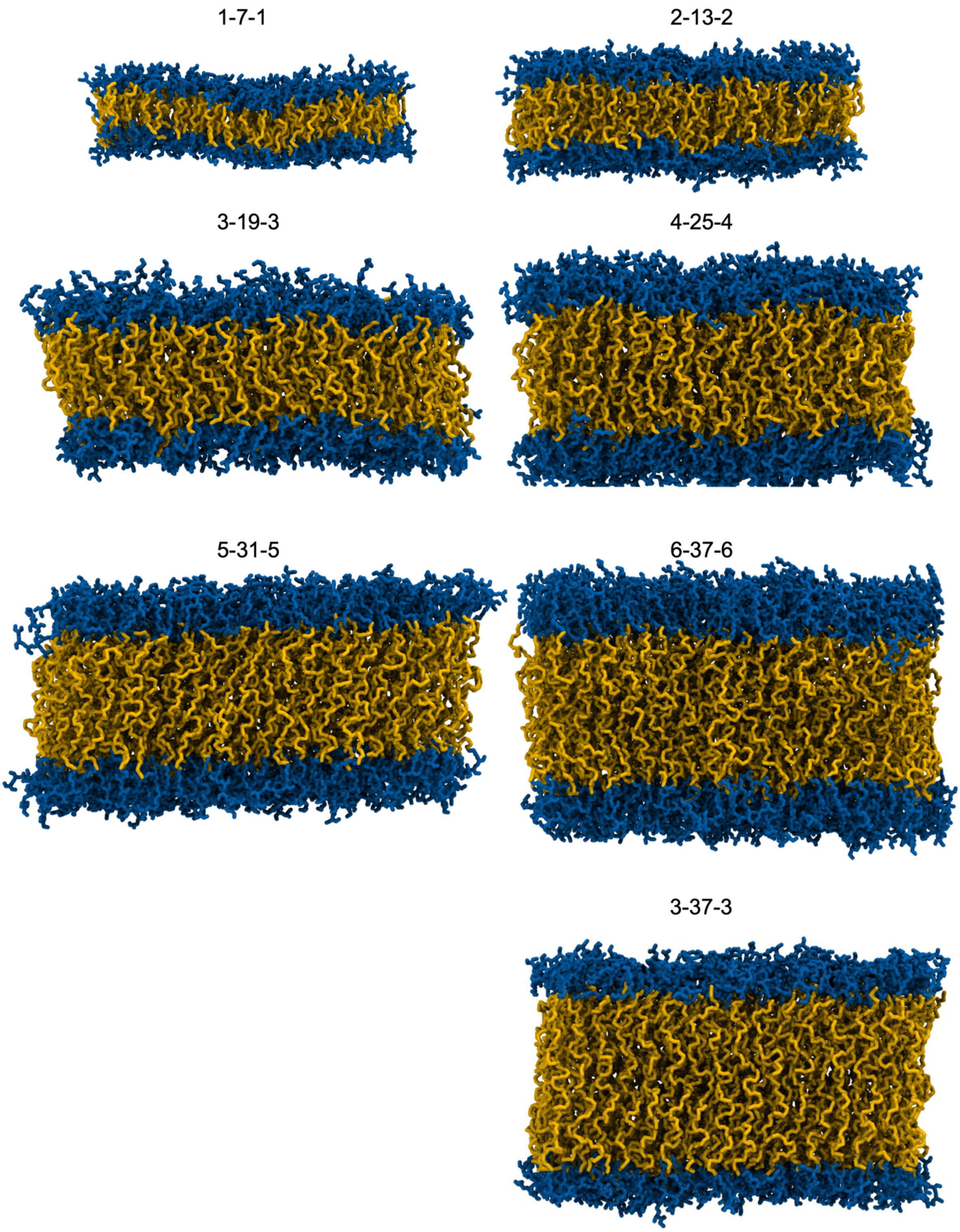

Figure S8. Atomistic models of polymersome membranes. Cross-section simulation snapshots of membranes showing PDMS in yellow and PMOXA in blue, water is removed for clarity. 2-13-2, 4-25-4, 6-37-6 serve as reference (from main Figure 3c).

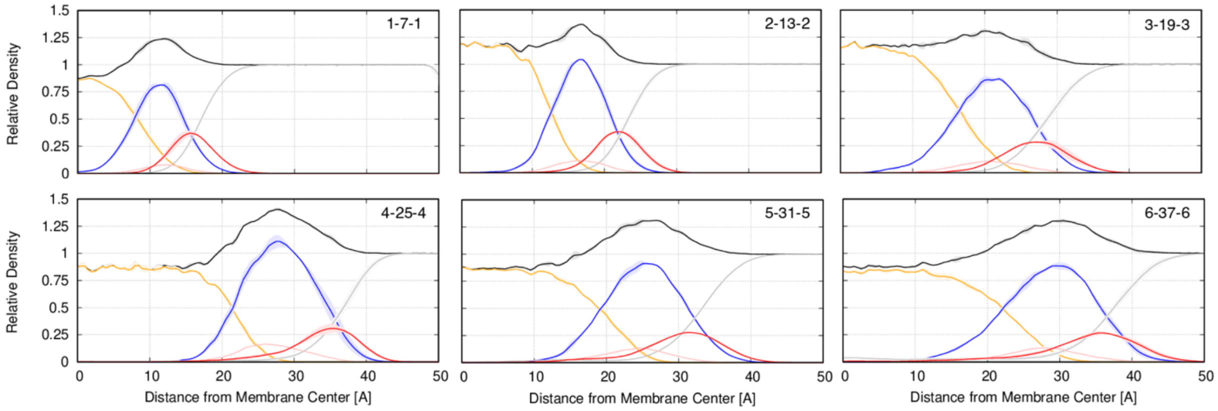

Figure S9. Atomistic molecular dynamics simulation of polymersome membranes. Cross-sectional densities of the membrane components (half of membranes from center shown; 1-7-1, 2-13-2, 3-19-3 (is reference point, from main Figure 3a), 4-25-4, 5-31-5, 6-37-6,) showing PDMS (yellow), PMOXA (blue), surface water (red), embedded water (pink), bulk water (gray), and total density (black).

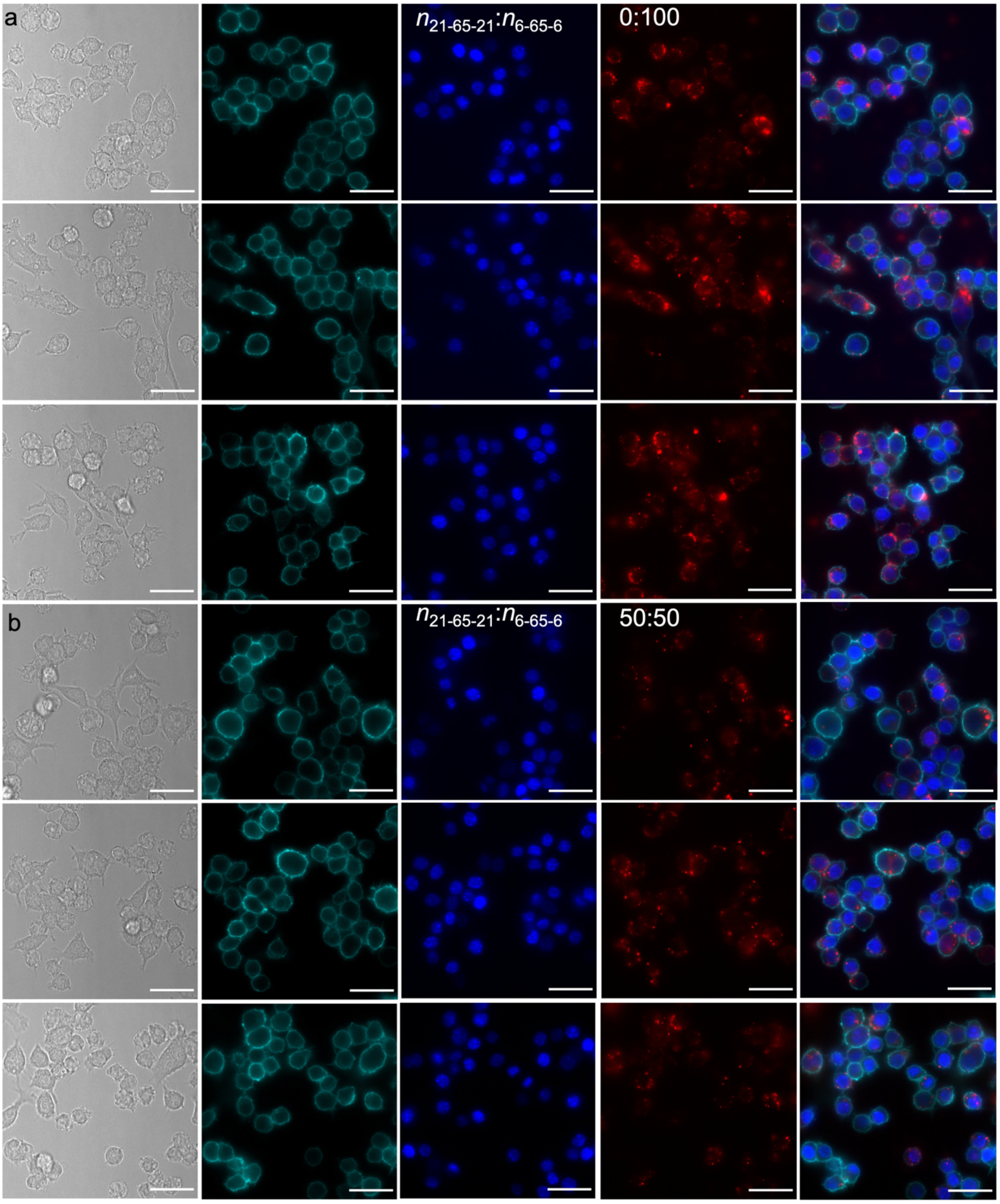

Figure S10. (a,b) Fluorescence widefield imaging of (a) 0:100 and (b) 50:50 ($n_{21\text{-}65\text{-}21}$:$n_{6\text{-}65\text{-}6}$, DiD labeled) polymersomes (red label, DiD, exposure kept constant for this channel for both samples) uptake in RAW 264.7 cells after 4 h incubation (cyan, WGA-488, membrane stain; blue, DAPI, nucleus). Scale bars, 30 μm.

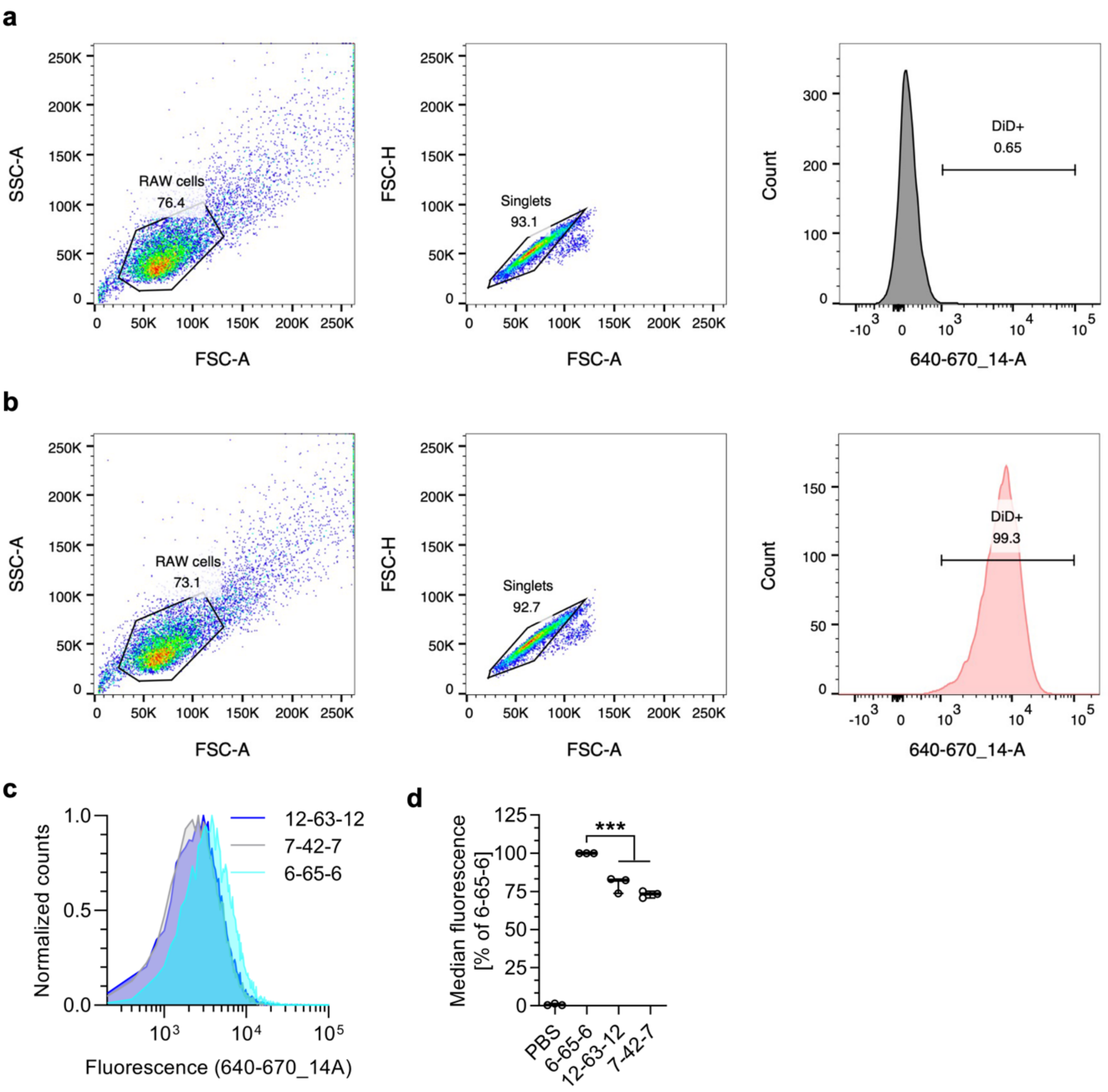

Figure S11. Cell association/uptake of polymersomes. (a,b) Flow cytometry gating strategy, examples are (a) PBS and (b) 6-65-6 polymersomes (DiD) uptake in RAW 264.7 cells. (c) Flow cytometry for polymersomes made from three different copolymers (DiD labeled) after 4 h incubation with RAW 264.7 cells. (d) Median fluorescence from (c) and independent repeats normalized to the 6-65-6 only sample. ($N = 3$ independent experiments, one-way ANOVA with Tukey's multiple comparisons test, ***$P < 0.001$).

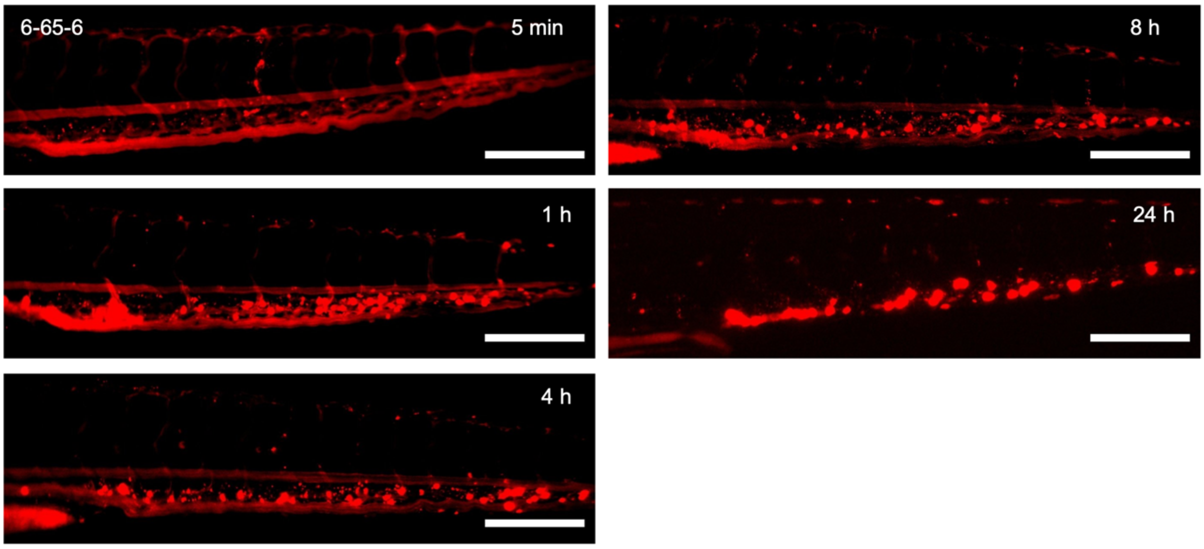

Figure S12. Circulation analysis in zebrafish embryos. Representative zebrafish embryo fluorescence images of the tail area after administration of polymersomes made from 6-65-6 (SRB loaded). Scale bars, 200 µm.

References

[1] F. Itel, M. Chami, A. Najer, S. Lörcher, D. Wu, I. A. Dinu, W. Meier, *Macromolecules* **2014**, *47*, 7588.
[2] S. Lörcher, W. Meier, *European Polymer Journal* **2017**, *88*, 575.
[3] S. Egli, Polymersomes for Biomedical Applications: Surface Functionalization of Silicone-Based Polymer Vesicles, **2011**.
[4] S. Thamboo, A. Najer, A. Belluati, C. von Planta, D. Wu, I. Craciun, W. Meier, C. G. Palivan, *Advanced Functional Materials* **2019**, *29*, 1.
[5] P. Müller, P. Schwille, T. Weidemann, *Bioinformatics* **2014**, *30*, 2532.
[6] P. Kapusta, P. GmbH, **2010**, 0.
[7] J. Penders, I. J. Pence, C. C. Horgan, M. S. Bergholt, C. S. Wood, A. Najer, U. Kauscher, A. Nagelkerke, M. M. Stevens, *Nature Communications* **2018**, *9*, 1.
[8] J. C. Phillips, D. J. Hardy, J. D. C. Maia, J. E. Stone, J. v. Ribeiro, R. C. Bernardi, R. Buch, G. Fiorin, J. Hénin, W. Jiang, R. McGreevy, M. C. R. Melo, B. K. Radak, R. D. Skeel, A. Singharoy, Y. Wang, B. Roux, A. Aksimentiev, Z. Luthey-Schulten, L. v. Kalé, K. Schulten, C. Chipot, E. Tajkhorshid, *Journal of Chemical Physics* **2020**, *153*, DOI 10.1063/5.0014475.
[9] K. Vanommeslaeghe, E. Hatcher, C. Acharya, S. Kundu, S. Zhong, J. Shim, E. Darian, O. Guvench, P. Lopes, I. Vorobyov, A. D. Mackerell, *Journal of Computational Chemistry* **2010**, *31*, 671.
[10] K. Vanommeslaeghe, E. P. Raman, A. D. MacKerell, *Journal of Chemical Information and Modeling* **2012**, *52*, 3155.
[11] W. Humphrey, A. Dalke, K. Schulten, *Journal of Molecular Graphics* **1996**, *14*, 33.
[12] G. J. Martyna, D. J. Tobias, M. L. Klein, *The Journal of Chemical Physics* **1994**, *101*, 4177.
[13] S. E. Feller, Y. Zhang, R. W. Pastor, B. R. Brooks, *The Journal of Chemical Physics* **1995**, *103*, 4613.
[14] F. De Chaumont, S. Dallongeville, N. Chenouard, N. Hervé, S. Pop, T. Provoost, V. Meas-Yedid, P. Pankajakshan, T. Lecomte, Y. Le Montagner, T. Lagache, A. Dufour, J. C. Olivo-Marin, *Nature Methods* **2012**, *9*, 690.

[15] N. J. K. Dal, A. Kocere, J. Wohlmann, S. Van Herck, T. A. Bauer, J. Resseguier, S. Bagherifam, H. Hyldmo, M. Barz, B. G. De Geest, F. Fenaroli, *Small* **2020**, *16*, 1906719.
